# Chemoenzymatic Synthesis of Indole-Containing Acyloin Derivatives

**DOI:** 10.3390/molecules28010354

**Published:** 2023-01-01

**Authors:** Saad Alrashdi, Federica Casolari, Aziz Alabed, Kwaku Kyeremeh, Hai Deng

**Affiliations:** 1 Department of Chemistry, University of Aberdeen, Aberdeen AB24 3UE, UK; 2 College of Science and Arts in Gurayat, Jouf University, King Khaled Road, Aljouf 42421, Saudi Arabia; 3 Marine and Plant Research Laboratory of Ghana, Department of Chemistry, University of Ghana, Legon, Accra P.O. Box LG56, Ghana

**Keywords:** indole, acyloin, chemoenzymatic synthesis, thiamine-diphosphate dependent enzymes, tryptophan, indole-3-pyruvate

## Abstract

Indole-containing acyloins are either key intermediates of many antimicrobial/antiviral natural products or building blocks in the synthesis of biologically active molecules. As such, access to structurally diverse indole-containing acyloins has attracted considerable attention. In this report, we present a pilot study of using biotransformation to provide acyloins that contain various indole substituents. The biotransformation system contains the tryptophan synthase standalone β-subunit variant, *Pf*TrpB^6^, generated from directed evolution in the literature; a commercially available L-amino acid oxidase (LAAO); and the thiamine-diphosphate (ThDP)-dependent enzyme NzsH, encoded in the biosynthetic gene cluster (*nzs*) of the bacterial carbazole alkaloid natural product named neocarazostatin A. The utilization of the first two enzymes, the *Pf*TrpB variant and LAAO, is designed to provide structurally diverse indole 3-pyruvate derivatives as donor substrates for NzsH-catalysed biotransformation to provide acyloin derivatives. Our results demonstrate that NzsH displays a considerable substrate profile toward donor substrates for production of acyloins with different indole ring systems, suggesting that NzsH could be further explored as a potential biocatalyst via directed evolution to improve the catalytic efficiency in the future.

## 1. Introduction

**Figure 1:**
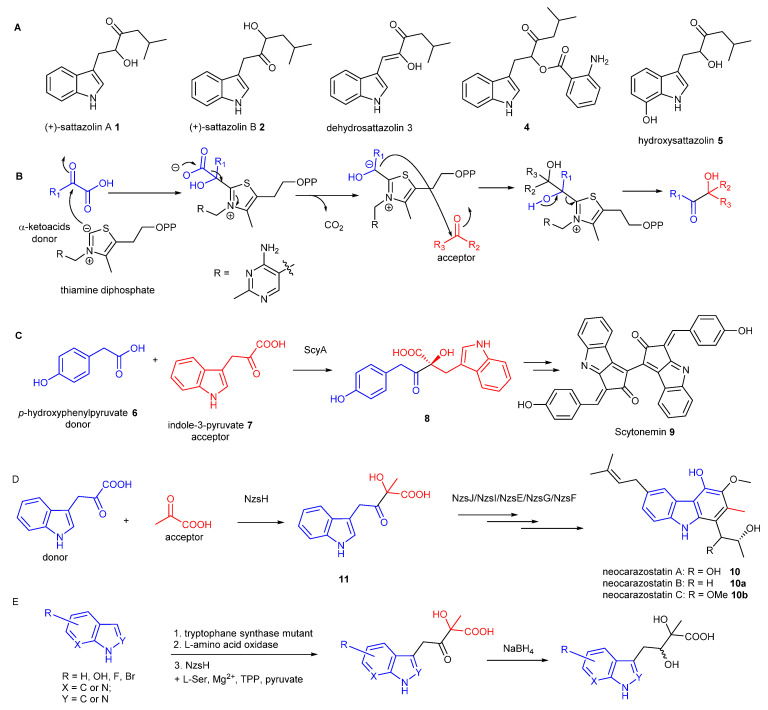
Indole-containing acyloins generated from ThDP-dependent enzymes. (**A**) indole-containing antimicrobial/antiviral acyloin natural products. (**B**) a generic mechanism of caboligation catalysed by ThDP-dependent enzymes. (**C**) The key biotransformation to generate indole-containing acyloin intermediate during the biosynthesis of scytonemin **9**. (**D**) The key biotransformation to generate indole-containing acyloin intermediate during the biosynthesis of neocarazostatin **10**. (**E**) The biotransformation to generate structurally diverse acyloins in this study.

Indole-containing acyloins are key precursor of antimicrobial/antiviral agents, such as sattazolins **1**–**5** (Figure 1A) [1,2,3]. They are also useful intermediates in synthesis of biologically active molecules such as carbazoles, β-carbolines and other indole-containing molecules [4]. However, access to structurally diverse indole-containing acyloins has presented a synthetic challenge.

Thiamine diphosphate (ThDP)-dependent enzymes are widely found in the biological systems and have been demonstrated to be involved in diverse biotransformation, including C–C, C–N, C–S, and C–O bond cleavage and formation [5]. The general mechanism underlying all these reactions is that the enzyme’s active site-mediated dissociation of the C2–H proton from the thiazolium ring of ThDP will generate the C2 anion ylid which is covalently bound to the donor substrate (Figure 1B). Subsequent decarboxylation results in the ThDP-bound enolate intermediate with nucleophilic reactivity which readily react with electrophilic acceptors, such as aldehydes, ketones or α-ketoacids to form the α-hydroxyl ketone (an acyloin) [6].

In many cases, pyruvate is the donor in the ThDP-dependent reactions. However, some of ThDP-dependent enzymes utilise a range of α-ketoacids to initiate carboligation reactions. For example, the ThDP dependent enzyme, ScyA, was found to couple *p*-hydroxyphenylpyruvate **6** as the donor and indole-3-pyruvate **7** as the acceptor to afford the β-ketoacid product **8**, the key intermediate during the biosynthesis of scytonemin **9**, a pigment produced by cyanobacteria [7]. During our biosynthetic studies of the bacterial carbazole alkaloid, neocarazostatin A **10** [8,9,10,11], we found that the ThDP-dependent enzyme NzsH catalyses an unusual carboligation between indole-3-pyruvate **7** as the donor and pyruvate as the acceptor to generate an indole-containing acyloin intermediate **11** [9], followed by further modifications to finally decorate **10** [10,11], consistent with a parallel study in the biosynthetic pathway of the bacterial carbazole analogue, carbazomycin [12]. NzsH only accepts 2-oxobutyrate, an analogue of pyruvate, as the acceptor, unlike other ThDP enzymes that normally display a considerable range of aldehyde and ketones [9]. Phylogenetic analysis demonstrated that NzsH formed a distinct clade with other ThDP-dependent enzymes, suggesting that NzsH represents a unique group of ThDP-dependent enzymes that utilises indole-3-pyruvate as the donor for the carboligation reaction [9]. However, the donor substrate plasticity of NzsH has remain elusive. More recently, two NzsH homologues, CbeiHKI805_0381 and Cbei2730, were shown to be responsible to produce the indole-containing acyloin core of antimicrobial/antiviral agents, sattazolins **1**-**5** and its derivatives, isolated from various strains of *Clostridium beijerinckii*, a bacterium used for industrial solvent production [13]. Interestingly, the enzymes can accept branched chain α-ketoacids as acceptor substrates and both pyruvate and indole-3-pyruvate as donor substrates [13].

Here, we report a pilot study of new three-enzyme coupled biotransformation to access indole-containing acyloin derivatives with structural diversity. The reaction mixture includes an engineered tryptophane synthase β-subunit, a commercially available L-amino acid oxidase (LAAO), and NzsH. This coupled reaction is initiated from commercially available indole or indole derivatives with L-Ser catalysed by the engineered tryptophane synthetase to provide tryptophane derivatives, followed by addition of LAAO for oxidation reactions to give the corresponding indole-3-pyruvate analogues. Inclusion of NzsH in the reactions finally allow access of a new range of indole-containing acyloin derivatives. This newly introduced functionalised indole-3-pyruvate analogues substantially broaden the donor substrate range of the ThDP-dependent NzsH enzyme, thus holding potential to generate new antiviral/antimicrobial acyloins with different indole ring systems.

## 2. Results and Discussion

**Figure 2:**
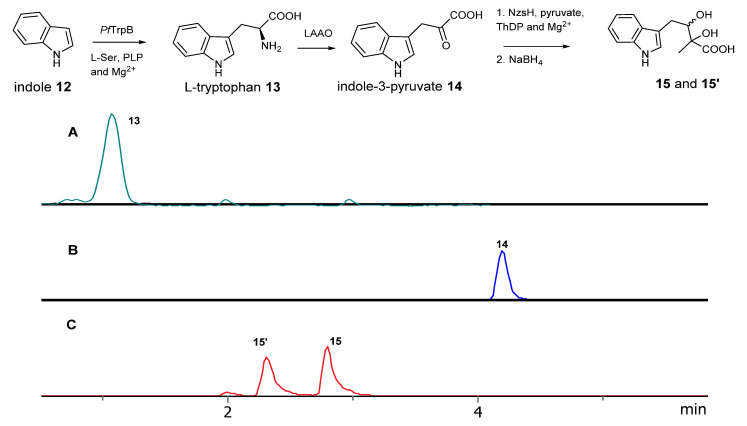
The reaction scheme of the step-wise coupled reactions leading the formation of indole-containing acyloins. (**A**) the extracted ion chromatography of L-tryptophan catalysed by the engineered *Pf*TrpB^6^. (**B**) the extracted ion chromatography of indole-3-pyruvate catalysed by a coupled reaction of the engineered *Pf*TrpB^6^ and L-AAO. (**C**) the extracted ion chromatography of reduced indole-containing acyloin derivative catalysed by a three-enzyme reaction of the engineered *Pf*TrpB^6^, L-AAO and NzsH, followed by NaBH_4_ reduction.

To generate structurally diverse indole-containing acyloin derivatives, indole-3-pyruvate derivatives with structural diversity to evaluate the substrate plasticity of NzsH is key. However, most of indole-3-pyruvate derivatives are not commercially available. In nature, indole-3-pyruvate is directly descended from L-tryptophane via deamination reactions. There has been a considerable interest in the application of tryptophane synthase to generate structurally diverse tryptophane derivatives due to its importance as a key building block for many bioactive molecules [14]. Tryptophan synthase is a heterodimeric complex consisting of two subunits, TrpA (α-subunit) catalysing the cleavage of indole glycerol phosphate to indole, and TrpB (β-subunit) mediating the coupling between indole and L-Ser to provide L-Tryptophan using pyridoxal phosphate (PLP) as cofactors (Supplementary Scheme S1A). The activities of both subunits are required for wild type tryptophan synthases, although only the reactivity of TrpB, coupling L-Ser and indole is necessary and desirable for L-tryptophan synthesis. Recent studies reported an engineered β-subunit of tryptophan synthase (*Pf*TrpB) from *Pyrococcus furiosus* as standalone function that restore the catalytic efficiency and surpass the activity of the native complex (Supplementary Scheme S1B) [15,16,17]. One variant (*Pf*TrpB^6^) with six amino acid site mutations displayed the best kinetics towards L-Ser and indole and a wide range of indole derivatives. To this end, we synthesized the gene coding *Pf*TrpB^6^ for overexpression in *E. coli*. *Pf*TrpB^6^ was expressed as an N-terminal pHis_6_ recombinant protein and was purified to near homogenesis by Ni-NTA chromatography, giving estimated molecular weight of ~45 kDa as previously reported (Supplementary Figure S1A). Incubation of *Pf*TrpB^6^ with indole, L-Ser and PLP resulted in the accumulation of L-tryptophan **13** as evidenced in our LC-MS and tandem MS analyses (Figure 2 and Supplementary Figure S2).

To further in situ generate indole-3-pyruvate from L-tryptophan, we decided to use commercially available L-amino acid oxidase (LAAO) from snake venom (Sigma Aldrich catalogue number: A5147) because this enzyme displays broad substrate tolerance toward amino acids and does not require any additional cofactors (Supplementary Scheme S1C). It also can efficiently shift the reaction equilibrium during the coupled reaction as observed in our previous report when the fluorination enzyme, FlA, from *Streptomyces cattleya*, was investigated for a chlorination reaction [18]. Addition of LAAO into the aforementioned enzyme system led to the formation of indole-3-pyruvate **14** as evidenced in our MS and tandem MS analysis (Figure 2 and Supplementary Figure S3). We next overexpressed the synthetic construct containing the gene *nzs*H in *E coli* BL-21 (DE3). NzsH was expressed as an N-terminal pHis_6_ recombinant protein and was purified to near homogenesis by Ni-NTA chromatography, giving estimated molecular weight of ~64 kDa as previously reported [9] (Supplementary Figure S1B). Inclusion of NzsH into the coupled reaction of *Pf*TrpB^6^ and LAAO together with TPP and Mg^2+^ resulted in the formation of the indole-containing acyloin scaffold. However, this molecule is not stable and is subjected to facile decarboxylation in the LC-MS analysis [9]. We used NaBH_4_ to reduce the β-ketone of the molecule to generate two diastereomeric diols, **15** and **15′**, as evidenced in our LC-MS and tandem MS analyses (Figure 2 and Supplementary Figure S4).

Encouraged by the results of this three-enzyme coupling system, we then acquired four commercially available indole derivatives including 4-fluoroindole **16**, 5-fluoroindole **17**, 4-bromoindole **18** and 6-hydroxyl-indole **19** (Table 1). Our LC-MS and tandem MS analysis demonstrated that all of these indole derivatives can be efficiently transformed into the corresponding indole-3-pyruvate derivatives, **26**–**29** as well as the corresponding diols, **31**–**35**, (Table 1), in the corresponding coupled chemoenzymatic systems.

**Figure 3:**
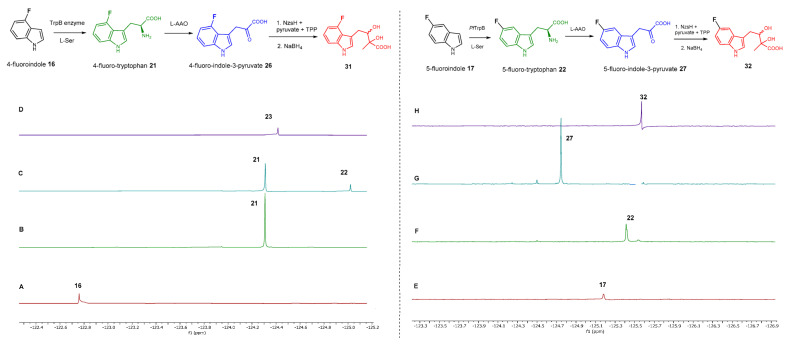
^13^F NMR results for the coupled reaction leading to the formation of 4-fluoroindole (left column) and 5-fluoroindole (right column) containing acyloins. (**A**) 4-fluoroindole (−122.8 ppm) as starting material. (**B**) 4-fluoro-L-tryptophan (−124.3 ppm) yielded from the *Pf*TrpB^6^ and 4-fluoroindole system. (**C**) 4-fluoroindole-3-pyruvate (−125.0 ppm) obtained when LAAO was added to the reaction. (**D**) 4-fluoro-acyloin (−124.4 ppm) generated by three-enzyme system. (**E**) 5-fluoroindole (−125.2 ppm) as starting material. (**F**) 5-fluoro-L-tryptophan (−125.4 ppm) yielded from the *Pf*TrpB^6^ and 4-fluoroindole system. (**G**) 5-fluoroindole-3-pyruvate (−124.8 ppm) obtained when LAAO was added to the reaction. (**H**) 5-fluoro-acyloin (−125.6 ppm) generated by the three-enzyme system.Azaindoles are structurally bioisosteric chemical structures to indoles in biological materials [19]. They display distinct physiochemical properties, aqueous solubility and total polar surface area due to the presence of extra nitrogen atom within the six membered ring in comparison to indoles. As such, azaindole-containing building blocks have attracted medicinal chemists to derive a number of pharmacological agents [20]. Among these, 7-azaindole **20** has appeared in a plethora of biologically active molecules [21], such as potent anticancer natural products, variolins [22] and their synthetic derivatives, meriolins [23]. To this end, we tested commercially available 7-azaindole **20** as the starting material in our biotransformation system. Again, the formation of aza-tryptophan **25**, azaindole-3-pyruvate **30** and azaindole-containing acyloin **35** were observed in the corresponding *Pf*TrpB^6^, *Pf*TrpB^6^ + LAAO and *Pf*TrpB^6^ + LAAO +NzsH systems, respectively, as evidenced in our LC-MS and MS tandem analyses (Table 1 and Supplementary Figures S9, S14 and S19). Taken together, NzsH display considerable substrate tolerance toward modified benzene rings of indole moieties when indole-3-pyruvate derivatives were used as substrates.

We also monitored the biotransformation of the formation of fluorinated indole-containing acyloins in ^19^F NMR. As shown in Figure 3A,E, the reaction was initiated by addition of 4-fluoroindole **16** (−122.8 ppm) and 5-fluoroindole **17** (−125.2 ppm), respectively, in the *Pf*TrpB^6^ -mediated systems to provide 4-fluoro-tryptophan **21** (−124.3 ppm) and 5-fluoro-tryptophan **22** (−125.4 ppm), respectively, in ^19^FNMR spectrum (Figure 3B,F). When LAAO was added to the reactions, 4-fluoro-indole-3-pyruvate **22** (−124.3 ppm) and 5-fluoroindole-3-pyruvate **27** (−124.8 ppm), respectively, appeared (Figure 3C,G). Finally, after inclusion of NzsH, the corresponding 4-fluoro-acyloin **31** (−124.4 ppm) and 5-fluoro-acyloin **32** (−125.6 ppm) was formed (Figure 3D,H), respectively.

**Figure 4:**
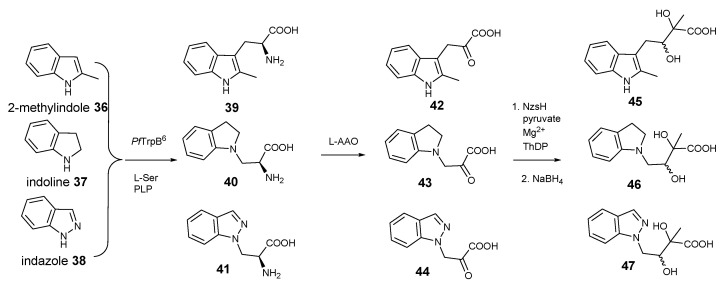
Coupled reactions yielding 2-methyl-indole, indoline and indazole-containing acyloins **45**–**47**, respectively, starting from three commercially available indole derivatives **36**–**38**.

Next, we acquired three commercially available indole derivatives, 2-methyl-indole **36**, indoline **37**, and indazole **38** that contain modified indole rings (Figure 4). Consistent with the previous report [15,16,17], the biotransformation catalysed by *Pf*TrpB^6^ gave the corresponding tryptophan derivatives, **39**–**41**, respectively. In all three cases, addition of LAAO provided the corresponding indole-pyruvate derivatives, **42**–**44**, respectively, further demonstrating the considerable substrate promiscuity of LAAO. To our surprise, these indole-pyruvate derivatives can be further utilized as the donor substrates in the NzsH-mediated enzymatic reaction to provide the final indole-containing acyloins, **45**–**47**, respectively, as observed in our LC-MS and tandem MS analyses of the corresponding reduced forms (Supplementary Figure S20–S28). Taken together, our studies demonstrated that NzsH, unlike its activities towards acceptor substrates, displays considerable substrate tolerances toward its donor substrates. However, further studies are required to improve the reactivities of NzsH enzyme toward the unnatural indole-3-pyruvate derivatives. This can be achieved via either identification of new NzsH homologues through comparative genomics or directed evolution to provide an engineered NzsH with the aim of finding a suitable biocatalyst with better kinetics in order to efficiently generate structurally diverse indole-containing acyloin derivatives.

In conclusion, we developed a coupled biotransformation system including a genetically modified β-subunit of tryptophan synthase (*Pf*TrpB^6^) from *Pyrococcus furiosus*, and a commercially available L-amino acid oxidase (LAAO) from snake venom and the ThDP-dependent NzsH enzyme from the biosynthetic pathway of neocarazostatins to access structurally diverse (aza)indole-containing acyloin derivatives. The combination of *Pf*TrpB^6^ and LAAO allowed generation of (aza)indole-3-pyruvate derivatives, starting from commercially available (aza)indole derivatives, which were subsequently accessed by NzsH-mediated reaction. Our results demonstrated that NzsH displays considerably good substrate tolerance, suggesting that this three-enzyme coupled system holds potential as a means of new biotransformation to produce indole-containing acyloins, important precursors of many biologically relevant molecules.

## 3. Methods and Materials

### 3.1. General Chemicals, Reagents, and Analytical Methods

All starting materials and reagents were bought from commercial sources and used as received. All biochemical reactions apart from where noted were carried out in triplicate. Before every set of measurements, triplicate control reactions were performed to ensure that the assay were functioning correctly. All flash column chromatography was carried out using silica purchased from Sigma Aldrich using the solvent system noted. ^19^F NMR spectra were recorded at 298 K on Bruker Avance III 400 using CFCl_3_ as an external reference. Chemical shifts are reported in parts per million (ppm) and coupling constants (*J*) are reported in Hertz (Hz).

Enzymatic assays were analyzed on a Bruker MaXis II ESI-Q-TOF-MS connected to an Agilent 1290 Infinity II UHPLC fitted with a Phenomenex Kinetex XB-C18 (2.6 μM, 100 × 2.1 mm) column. The column was eluted with a linear gradient of 5–100% MeCN containing 0.1% formic acid over 15 min. The mass spectrometer was operated in positive ion mode with a scan range of 200–3000 m/z. Source conditions were: end plate offset at −500 V; capillary at −4500 V; nebulizer gas (N_2_) at 4.0 bar; dry gas (N_2_) at 9.0 L min^−1^; dry temperature at 200 °C. Ion transfer conditions were: ion funnel RF at 400 Vpp; multiple RF at 200 Vpp; quadrupole low mass at 200 *m*/*z*; collision energy at 8.0 eV; collision RF at 2000 Vpp; transfer time at 110.0 μs; pre-pulse storage time at 10.0 μs. MS data were analysed using Bruker DataAnalysis or Thermo Xcalibur.

### 3.2. General Methods of Protein Expression and Purification

The synthetic constructs encoding *Pf*TrpB^6^ and NzsH proteins were purchased from Genscript Ltd. and were individually transformed into *E. coli* BL21 (DE3). Single colonies from each transformation were grown overnight in LB media (5 mL) containing kanamycin (50 µg/mL) and chloramphenicol (25 µg/mL). The overnight culture was transferred to fresh LB medium (500 mL) supplemented with kanamycin (50 µg/mL) and cultivated at 37 °C until the cell density reached an OD_600_ of 0.6. Isopropyl β-D-1-thiogalactopyranoside (IPTG) was added to a final concentration of 0.1 mM to induce protein expression. Cells were grown for 16–20 h at 16 °C and then harvested by centrifugation at 4 °C. The cells pellets were resuspended in ice-cold lysis buffer (20 mM Tris-HCl, 300 mM NaCl, 10 mM imidazole, pH 8.0), and further disrupted by Ultrasonic Homogenizer JY92-IIN. Then, the supernatant of cell debris was loaded onto Ni-NTA-affinity column. Bound proteins were eluted with the same Tris-HCl buffer containing different concentrations of imidazole. The desired elution fractions were combined and concentrated using a Centrifugal Filter Unit (Millipore). The final yields of *Pf*TrpB^6^ and NzsH were estimated to be 10 mg/100 mL culture and 5 mg/100 mL culture, respectively.

### 3.3. Biochemical Reactions

A sample of *Pf*TrpB^6^ (20 µM) was incubated with indole or indole derivatives (1 mM), L-Ser (1 mM) and PLP (1 mM) in phosphate buffer (50 mM, pH 7.5) to the final volume of 100 µL at 28 °C for 3 h and then quenched by addition of 100 µL of acetonitrile. The mixture was centrifuged at 13,000 rpm for 10 min to remove protein precipitates.

For the production of indole-3-pyruvate derivatives, a sample of *Pf*TrpB^6^ (20 µM) was incubated with indole or indole derivatives (1 mM), L-Ser (1 mM) and PLP (1 mM) in phosphate buffer (50 mM, pH 7.5) to a final volume of 50 µL at 28 °C for 3 h. To this mixture were added LAAO (20 µM) to a final volume of 100 µL at 28 °C for another 1 h. The reaction mix was then quenched by addition of 100 µL of acetonitrile. The mixture was centrifuged at 13,000 rpm for 10 min to remove protein precipitates.

For the production of indole-containing acyloin derivatives, a sample of *Pf*TrpB^6^ (20 µM) was incubated with indole or indole derivatives (1 mM), L-Ser (1 mM) and PLP (1 mM) in phosphate buffer (50 mM, pH 7.5) to a final volume of 100 µL at 28 °C for 3 h. To this mixture were added LAAO (20 µM) to a volume of 100 µL at 28 °C for another 1 h. Inclusion of NzsH (25 µM), TPP (1 mM) and Mg^2+^ (1 mM) into the mixture was perform to the final volume of 100 µL at 28 °C for 3 h. Because of the instability of acyloins, overdose NaBH_4_ was treated to the three-enzyme systems. Finally, the reaction mix was quenched by addition of 100 µL of acetonitrile. The mixture was centrifuged at 13,000 rpm for 10 min to remove protein precipitates.

All the supernatants from the above reaction systems were then analyzed by Bruker MaXis II QTOF in tandem with an Agilent 1290 Infinity UHPLC. Samples were separated on a Phenomenex Kinetex XB-C18 (2.6 μM, 100 × 2.1 mm) column with a mobile phase of 5% ACN + 0.1% formic acid to 100% ACN + 0.1% formic acid in 15 min.

## Figures and Tables

**Table 1:** Structures of indole derivatives and related indole-containing products generated from the step-wise coupled reaction. Addition of NzsH together with ThDP and Mg^2+^ provided the corresponding indole-containing acyloin derivatives, **31**–**35** (Table 1), as evidenced in our LC-MS and tandem MS analyses (Supplementary Figure S5–S19).

Indole Derivatives	Tryptophan Derivatives	Indole-3-Pyruvate Derivatives	Acyloin Derivatives
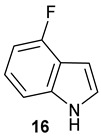	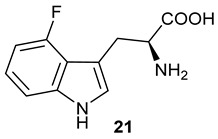	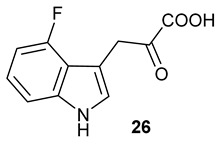	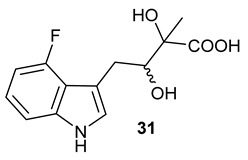
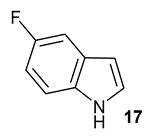	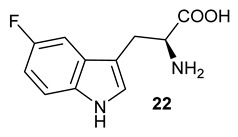	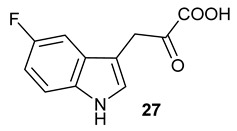	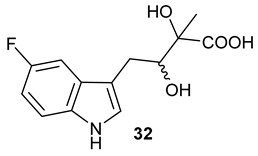
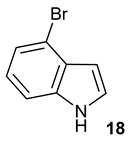	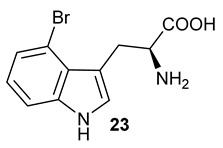	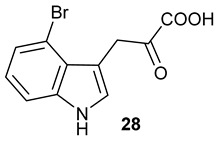	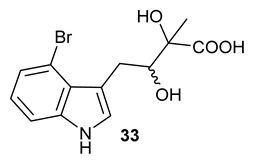
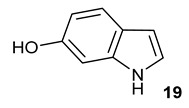	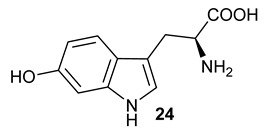	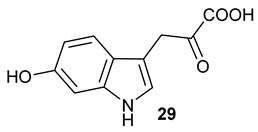	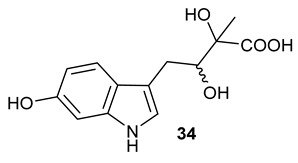
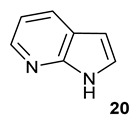	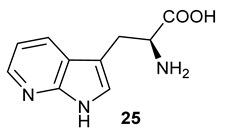	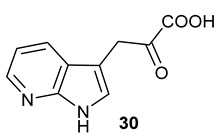	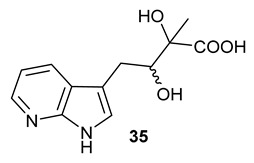

## Data Availability

Data are contained within the article.

## Supplementary Materials

The following supporting information can be downloaded at: https://www.mdpi.com/article/10.3390/molecules28010354/s1, Figure S1 SDS Page electrophoresis analysis of purifies PfTrpB6(A) and NzsH(B); Figure S2. LC-MS (A) and MS/MS data (B) of L-tryptophan **13**.;Figure S3. LC-MS (A) and MS/MS data (B) of indole-3-pyruvate **14**. Figure S4. LC-MS (A) and MS/MS data(B) of indole-containing acyloin **15**.Figure S5. LC-MS (A) and MS/MS data (B) of 4-fluoro-indole-tryptophan **21**. Figure S6. LC-MS (A) and MS/MS data (B) of 5-fluoro-indole-tryptophan **22**. Figure S7. LC-MS (A) and MS/MS data (B) of 4-bromo-indole-tryptophan **23.** Figure S8. LC-MS (A) and MS/MS data (B) of 6-hydroxyl-tryptophan **24**.Figure S9. LC-MS (A) and MS/MS data (B) of 7-azaindole-tryptophan **25**.Figure S10. LC-MS (A) and MS/MS data (B) of 4-fluoro-3-indole pyruvate **26;** Figure S11. LC-MS (A) and MS/MS data (B) of 5-fluoro-3-indole pyruvate **27.** Figure S12. LC-MS (A) and MS/MS data (B) of 4-bromo-3-indole pyruvate **28**. Figure S13. LC-MS (A) and MS/MS data (B) of 6-hydroxyl-3-indole pyruvate **29**. Figure S14. LC-MS (A) and MS/MS data (B) of 7-azaindole-3-pyruvate **30**. Figure S15. LC-MS (A) and MS/MS data (B) of 4-fluoro-indole-containing acyloin **31**. Figure S16. LC-MS (A) and MS/MS data (B) of 5-fluoro-indole-containing acyloin **32**.Figure S17. LC-MS (A) and MS/MS data (B) of 4-bromo-indole-containing acyloin **33;** Figure S18. LC-MS (A) and MS/MS data (B) of 6-hydroxyl-indole-containing acyloin **34**.Figure S19. LC-MS (A) and MS/MS data (B) of 7-azaindole-containing-acyloin **35**.Figure S20. LC-MS (A) and MS/MS data (B) of 2-methyl-indole-tryptophan **39**.Figure S21. LC-MS (A) and MS/MS data (B) of indoline-tryptophan **40**. Figure S22. LC-MS (A) and MS/MS data (B) of indazole-tryptophan **41**. Figure S23. LC-MS (A) and MS/MS data (B) of 2-methyl-indole-3-pyruvate **42**.Figure S24. LC-MS (A) and MS/MS data (B) of indoline-3-pyruvate **43**.Figure S25. LC-MS (A) and MS/MS data (B) of indazole-3-pyruvate **44**.Figure S26. LC-MS (A) and MS/MS data (B) of 2-methyl-indole-containing-acyloin **45**.Figure S27. LC-MS (A) and MS/MS data (B) of indoline-containing-acyloin **46**.Figure S28. LC-MS (A) and MS/MS data (B) of indazole-containing-acyloin **47**.

## Sample Availability

Samples of the compounds are available from the authors after request.
