# Supplementary material for: Chemoenzymatic Synthesis of Indole-Containing Acyloin Derivatives
Source: Molecules. 2023 Jan 1;28(1):354. doi: 10.3390/molecules28010354 (PMC9822442; doi:10.3390/molecules28010354)
Supplement: Supplementary file 1 [file molecules-28-00354-s001.zip › molecules-2089038-supplementary.pdf]

Supplementary Information

Chemoenzymatic synthesis of indole-containing acyloin derivatives

Saad Alrashdi [1, 2], Federica Casolari[1#], Aziz Alabed [1#], Kwaku Kyeremeh[3], Hai Deng [1]

1. Department of Chemistry University of Aberdeen Aberdeen AB24 3UE Scotland, United Kingdom
2. College of Science and Arts in Gurayat, Jouf University, King Khaled Road, Kingdom of Saudi Arabia
3. Marine and Plant Research Laboratory of Ghana, Department of Chemistry, University of Ghana, P.O. Box LG56, Legon-Accra, Ghana

Corresponding author: h.deng@abdn.ac.uk

[#]: these authors contributed equally.

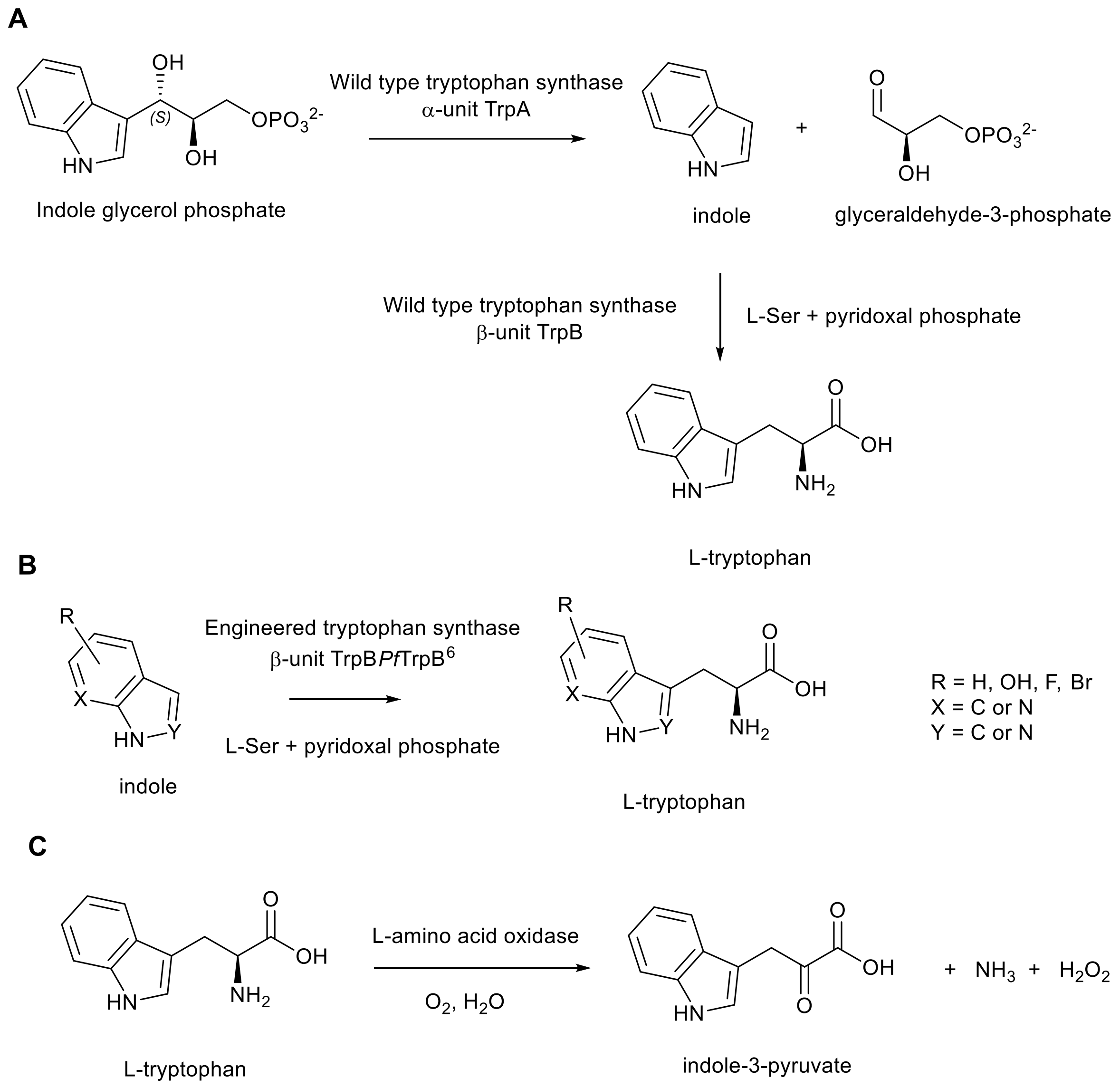

Scheme S1. The reactions of indole and L-Ser catalysed by wild type tryptophane synthase a- and b-units (**A**), the engineered tryptophan synthase b-unit *Pf*TrpB6 (**B**) and the commercially available L-amino acid oxidase (**C**).

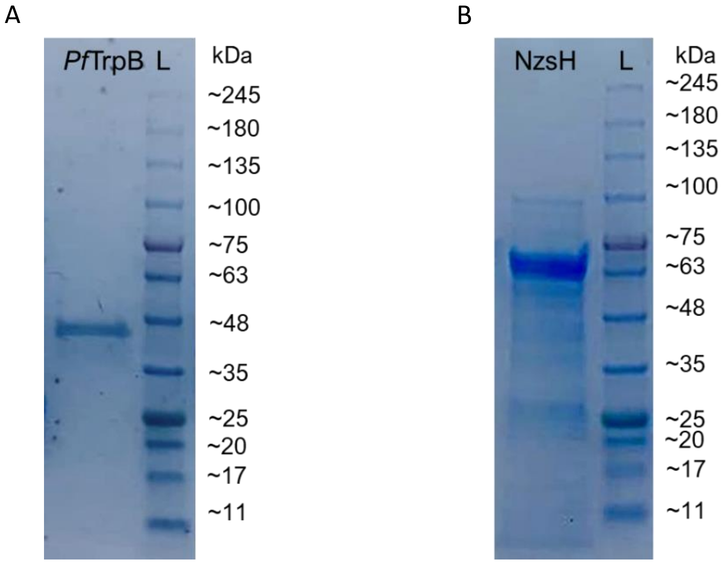

Figure S1. SDS Page electrophoresis analysis of purifies *Pf*TrpB[6](A) and NzsH(B).

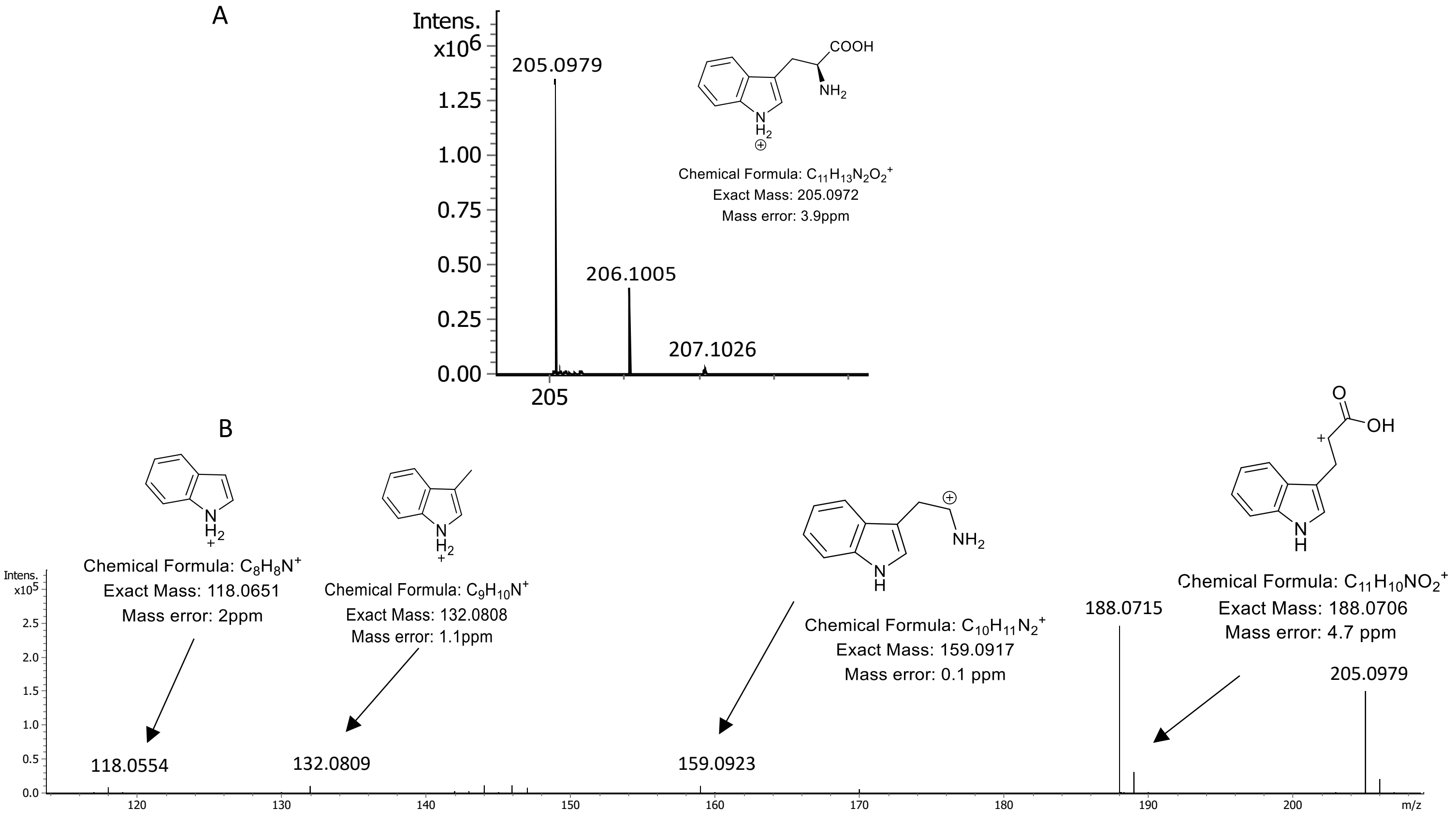

Figure S2. LC-MS (A) and MS/MS data (B) of L-tryptophan **13**.

A

B

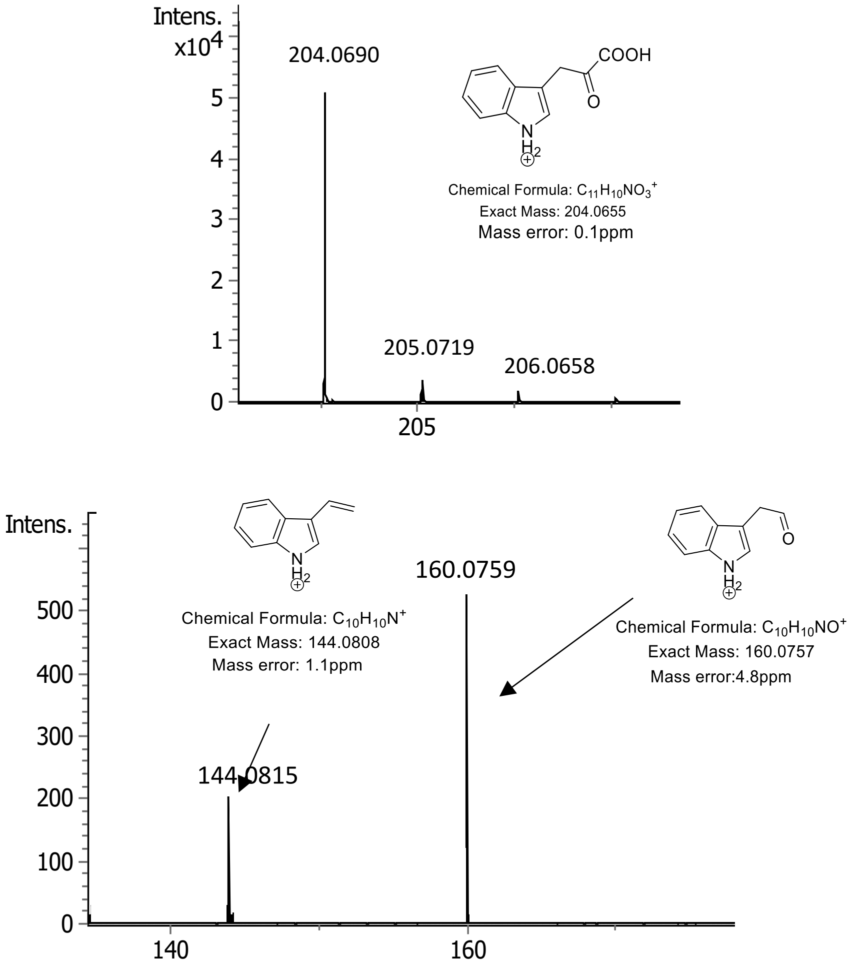

Figure S3. LC-MS (A) and MS/MS data (B) of indole-3-pyruvate **14**.

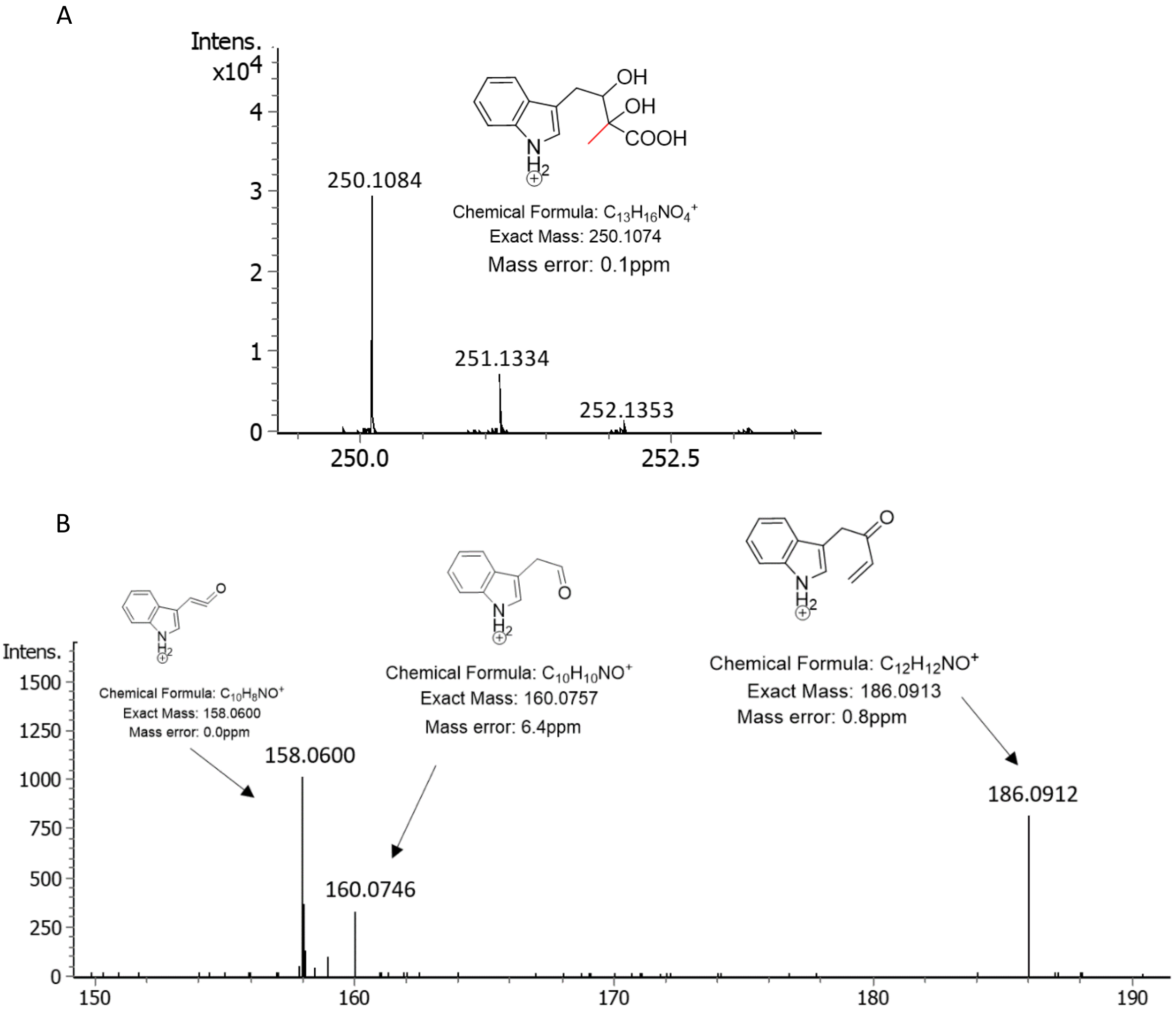

Figure S4. LC-MS (A) and MS/MS data(B) of indole-containing acyloin **15**.

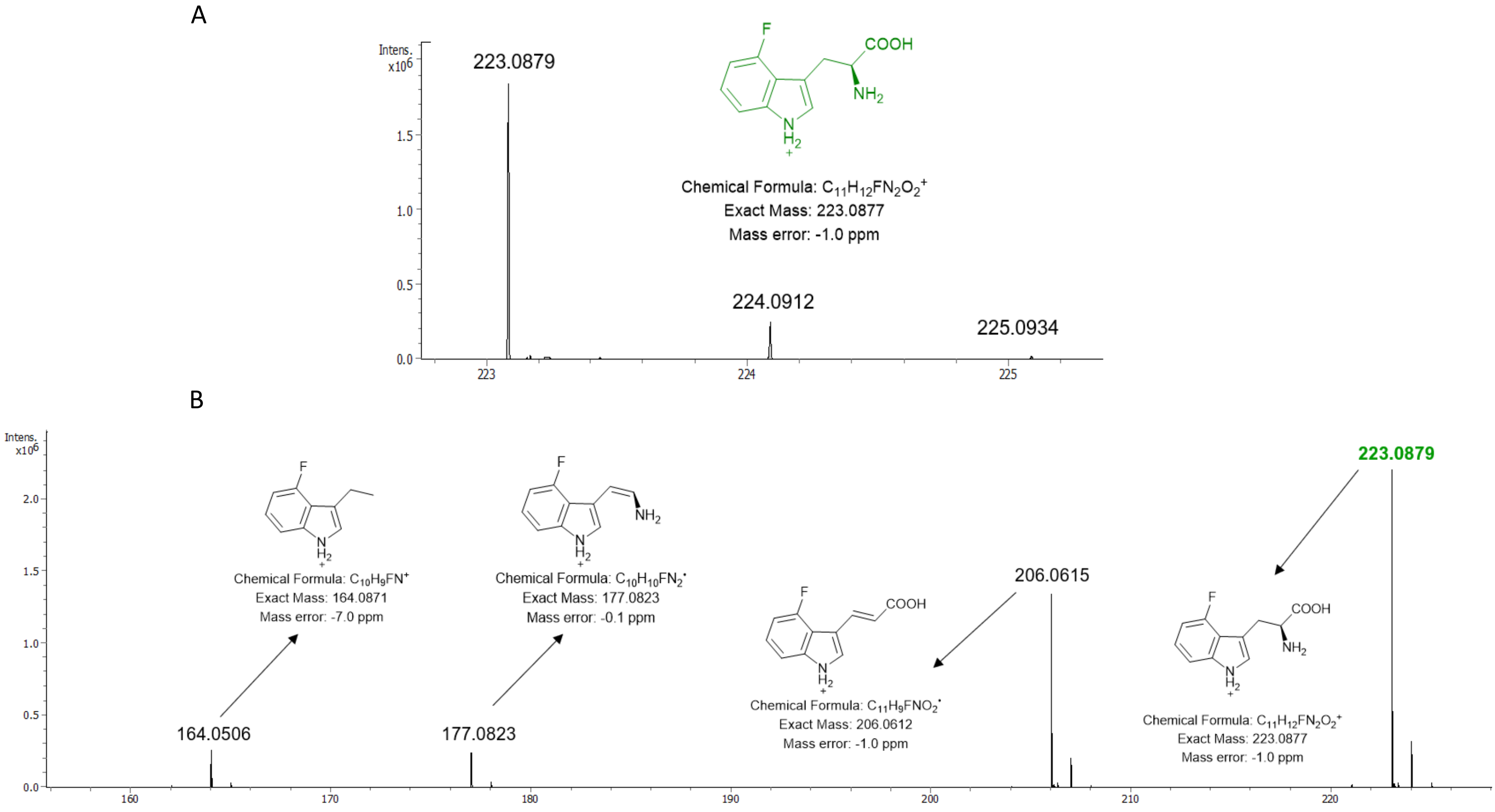

Figure S5. LC-MS (A) and MS/MS data (B) of 4-fluoro-indole-tryptophan **21**.

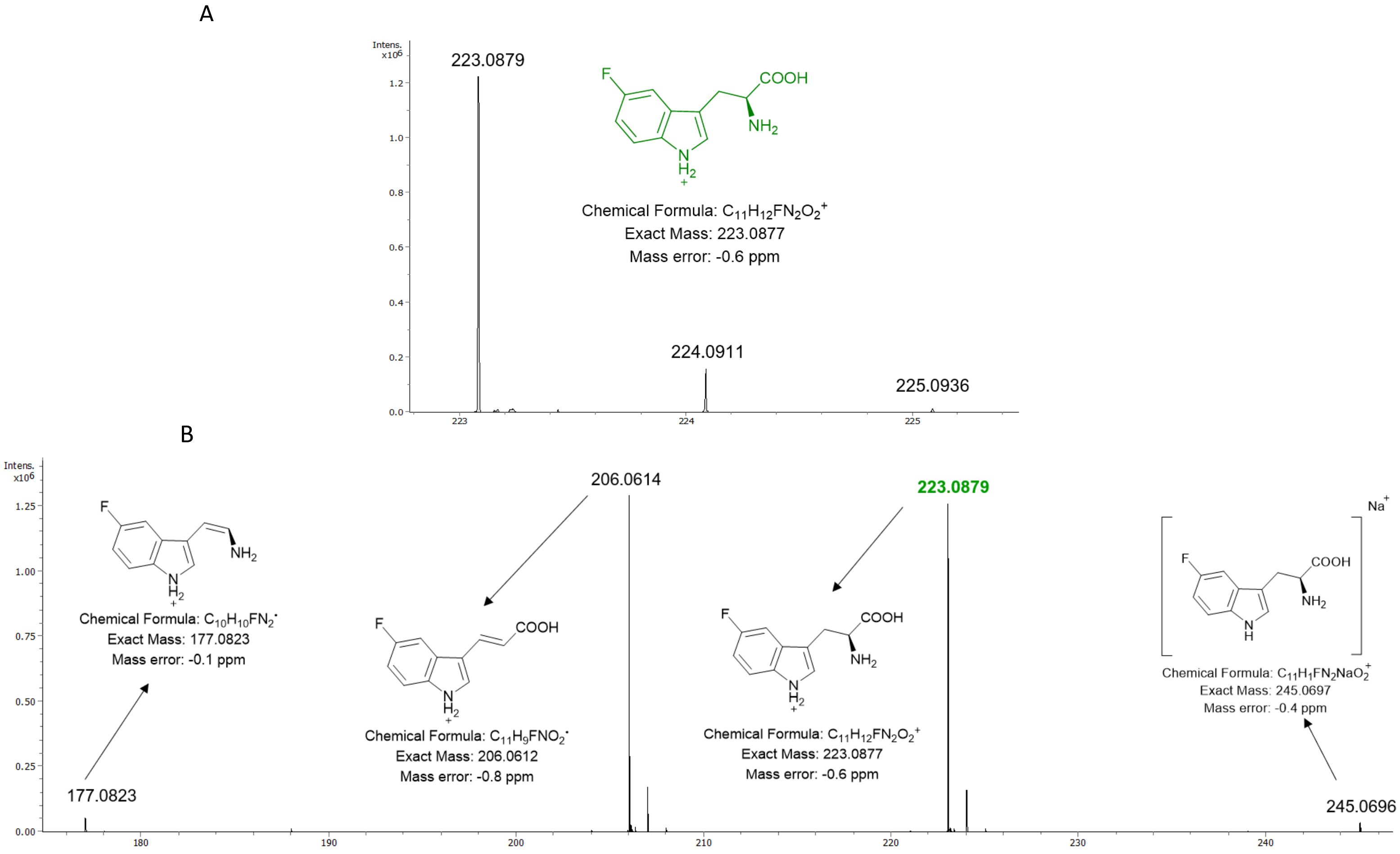

Figure S6. LC-MS (A) and MS/MS data (B) of 5-fluoro-indole-tryptophan **22**.

A

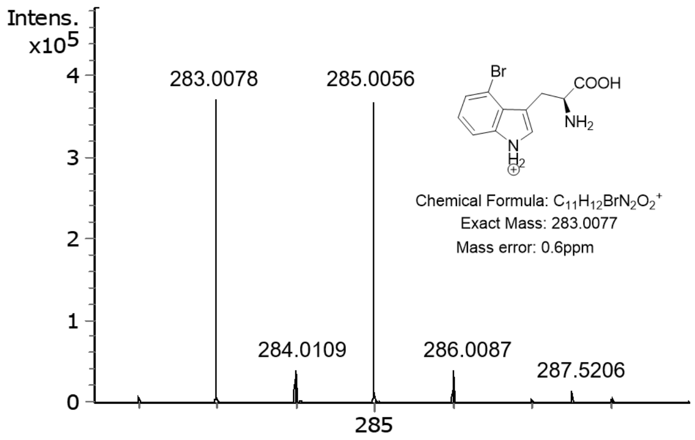

B

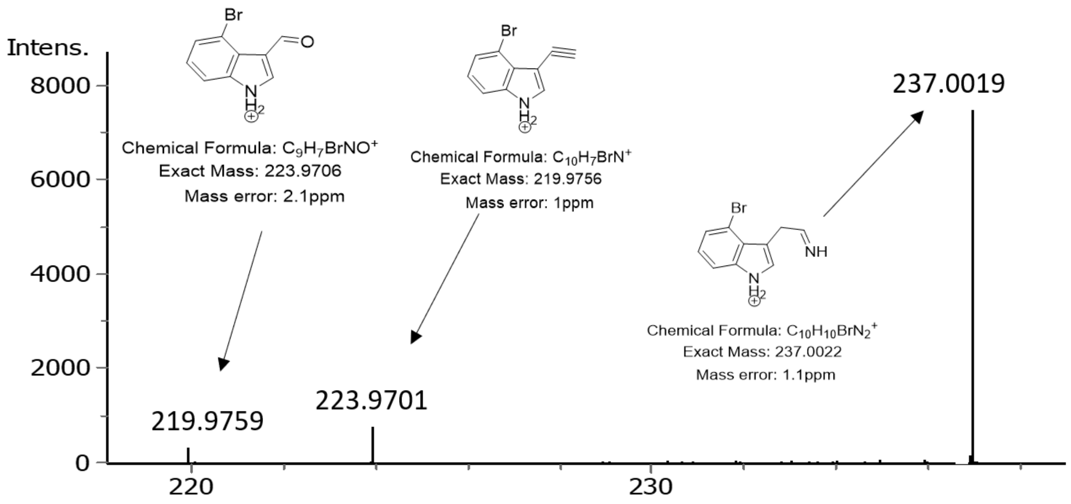

Figure S7. LC-MS (A) and MS/MS data (B) of 4-bromo-indole-tryptophan **23**.

A

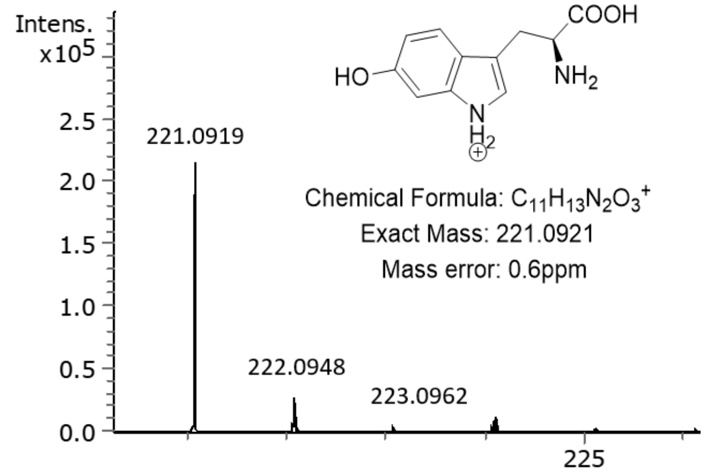

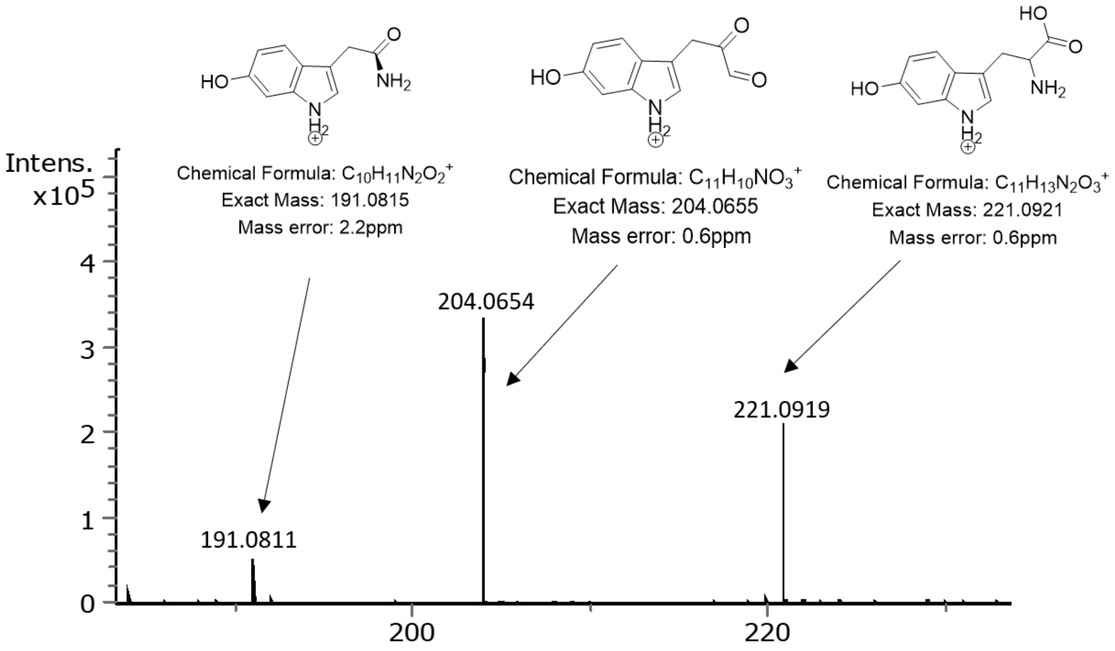

Figure S8. LC-MS (A) and MS/MS data (B) of 6-hydroxyl-tryptophan **24**.

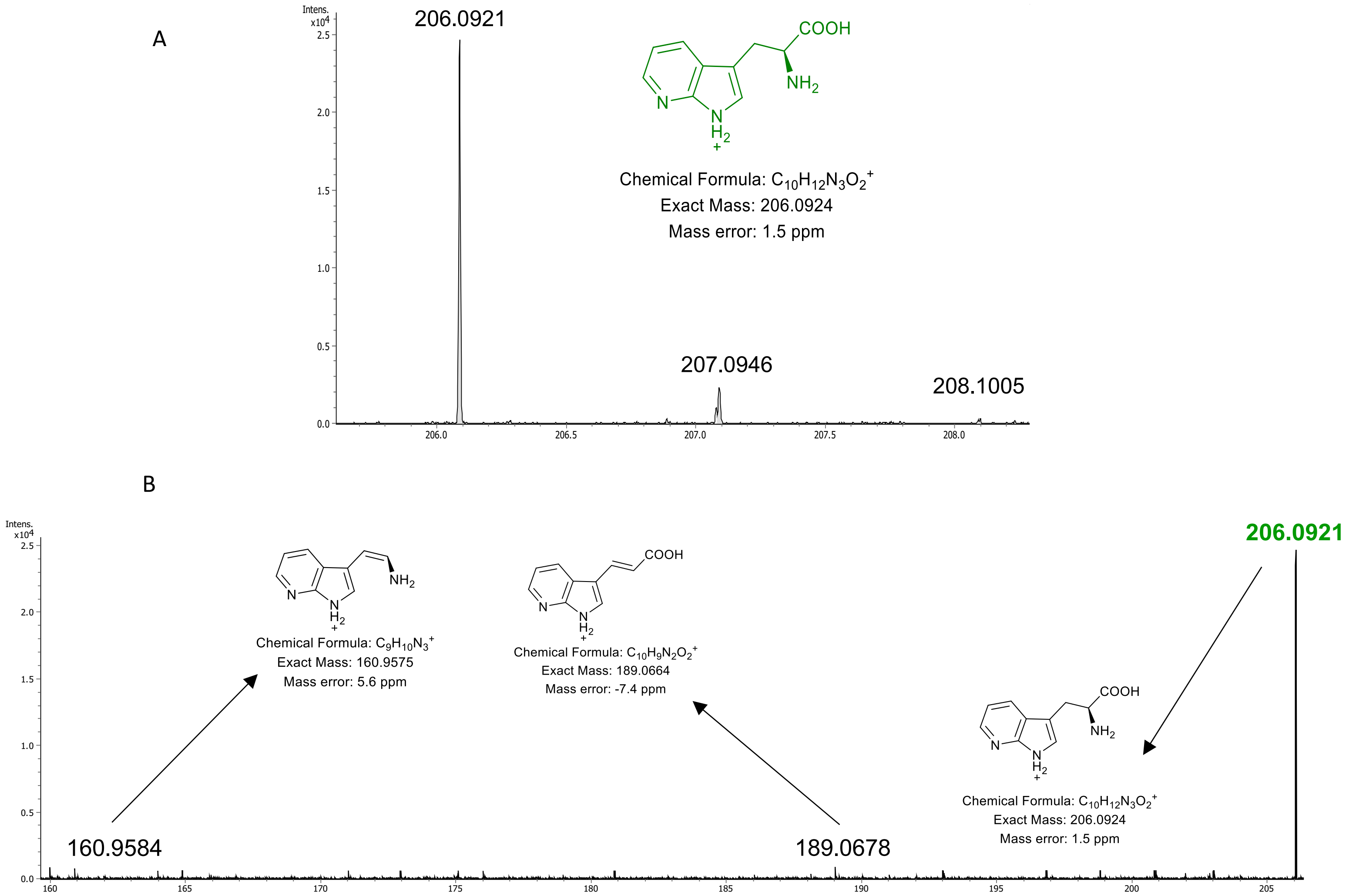

Figure S9. LC-MS (A) and MS/MS data (B) of 7-azaindole-tryptophan **25**.

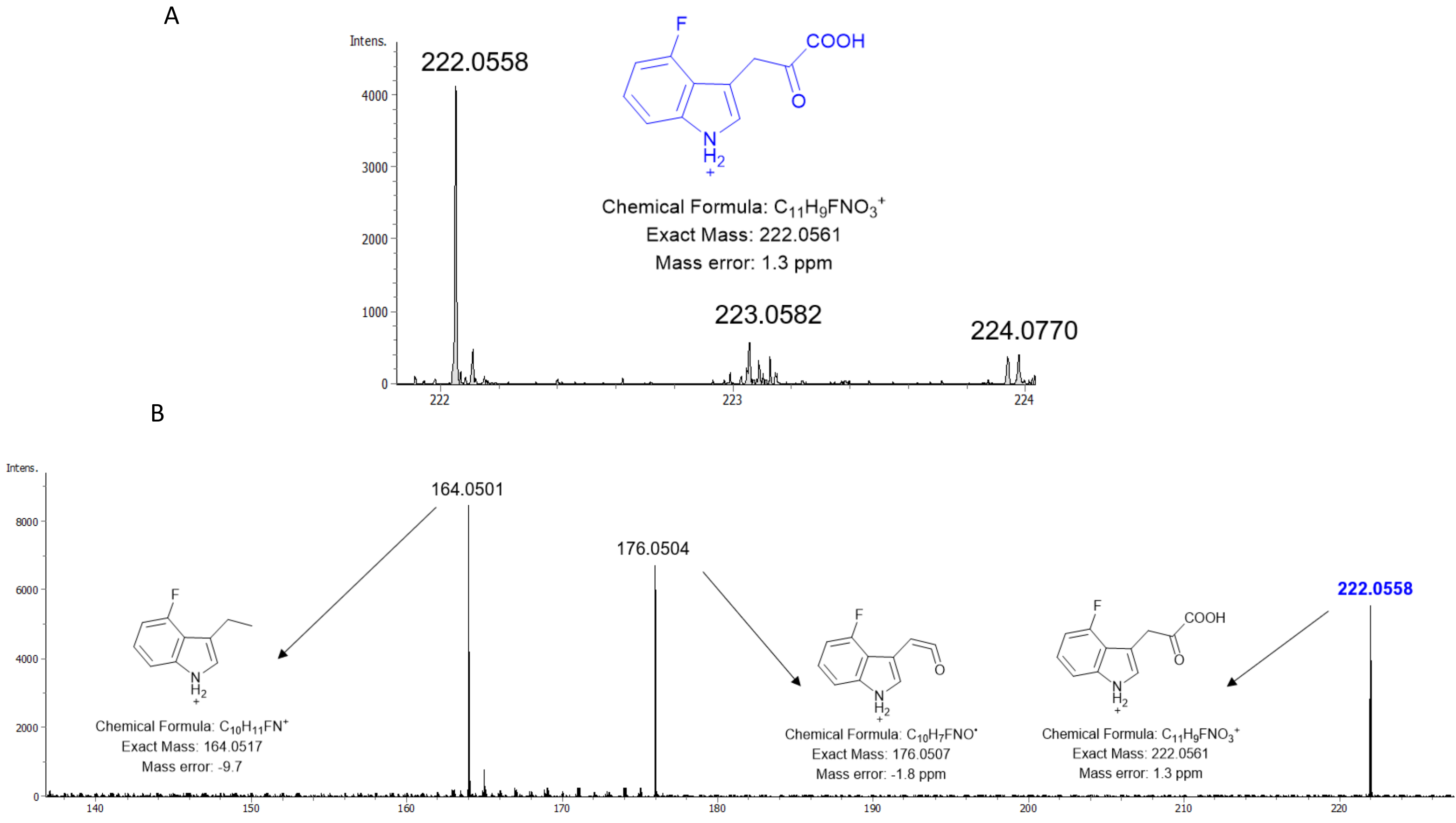

Figure S10. LC-MS (A) and MS/MS data (B) of 4-fluoro-3-indole pyruvate **26**.

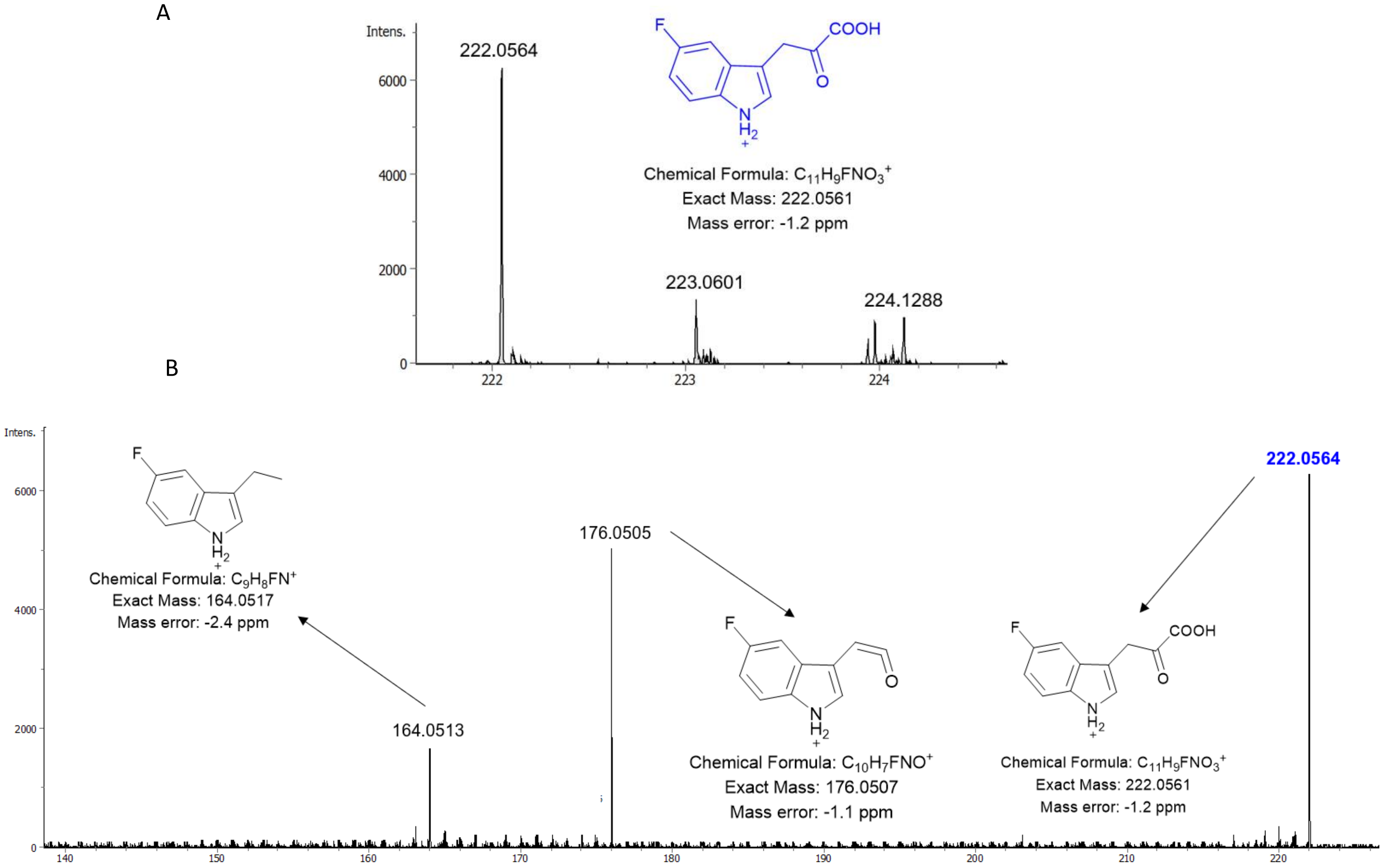

Figure S11. LC-MS (A) and MS/MS data (B) of 5-fluoro-3-indole pyruvate **27**.

A

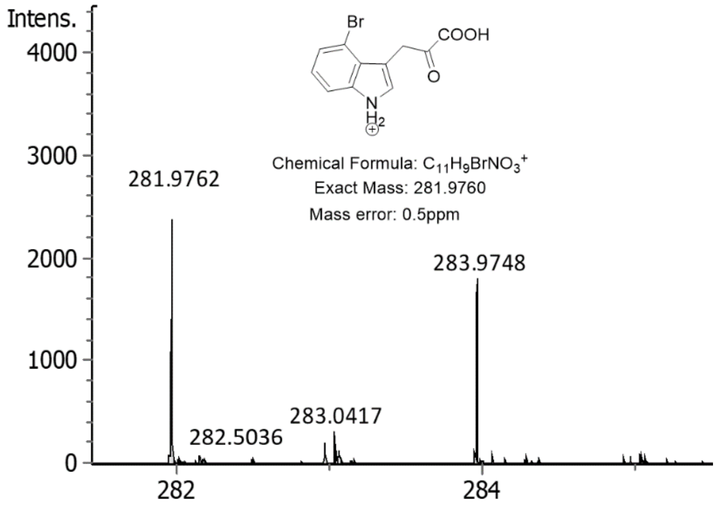

B

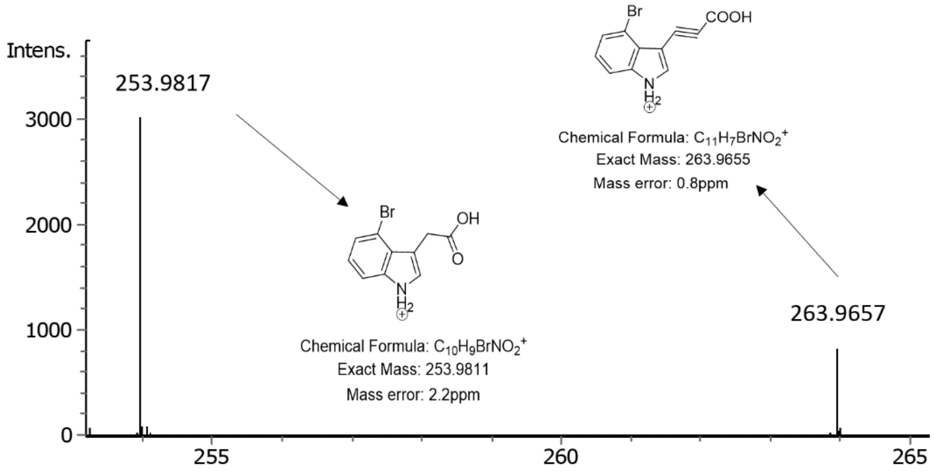

Figure S12. LC-MS (A) and MS/MS data (B) of 4-bromo-3-indole pyruvate **28**.

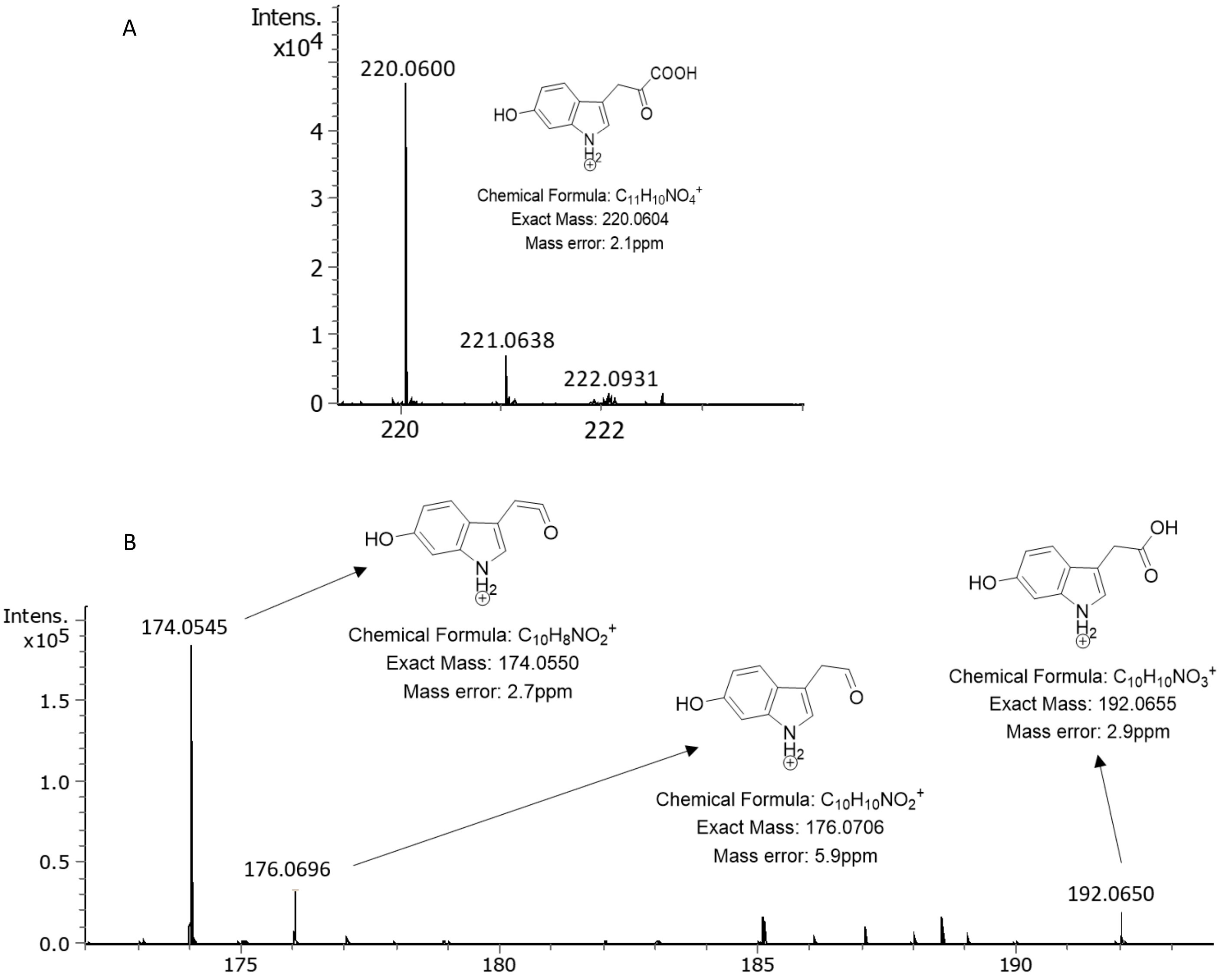

Figure S13. LC-MS (A) and MS/MS data (B) of 6-hydroxyl-3-indole pyruvate **29**.

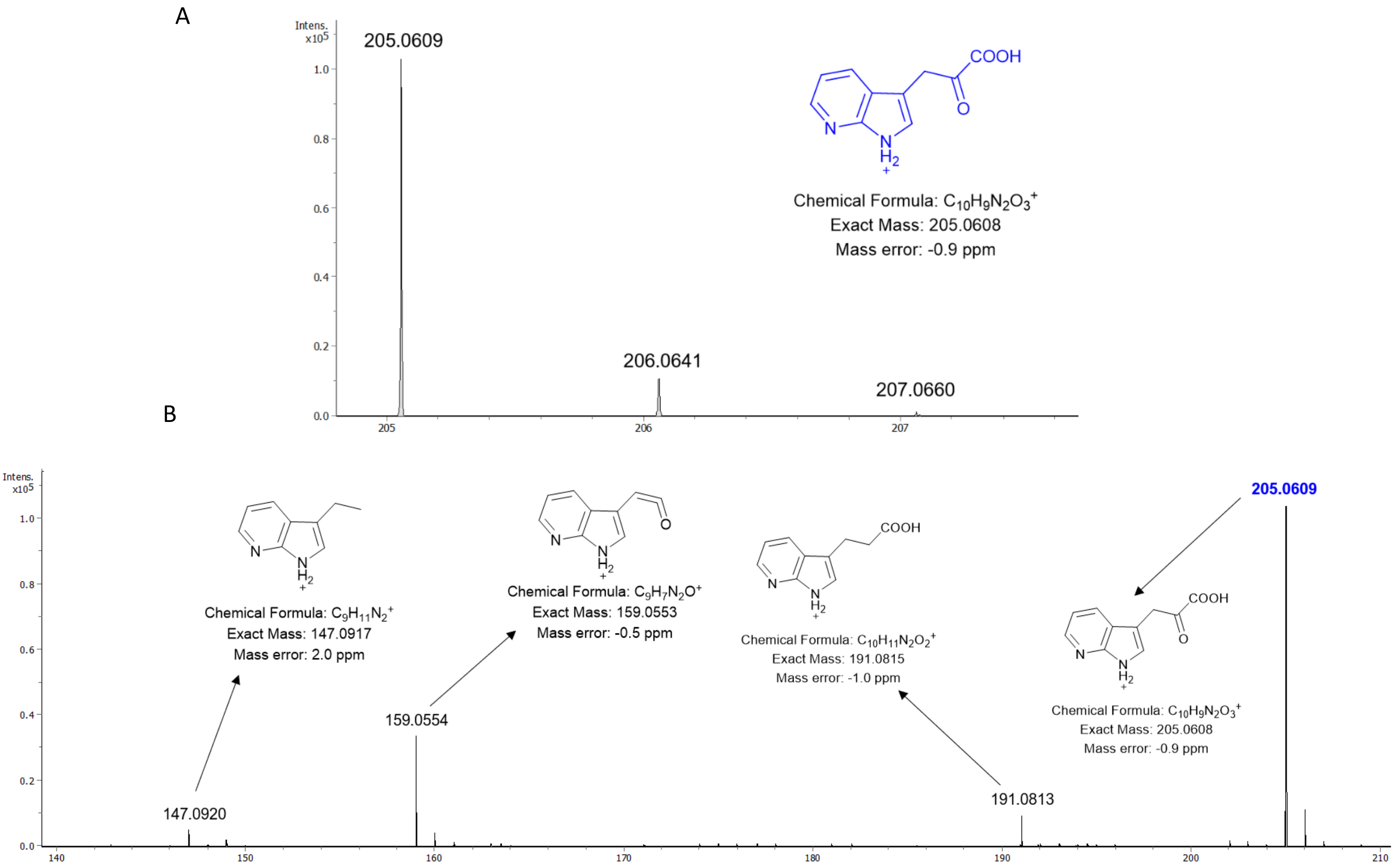

Figure S14. LC-MS (A) and MS/MS data (B) of 7-azaindole-3-pyruvate **30**.

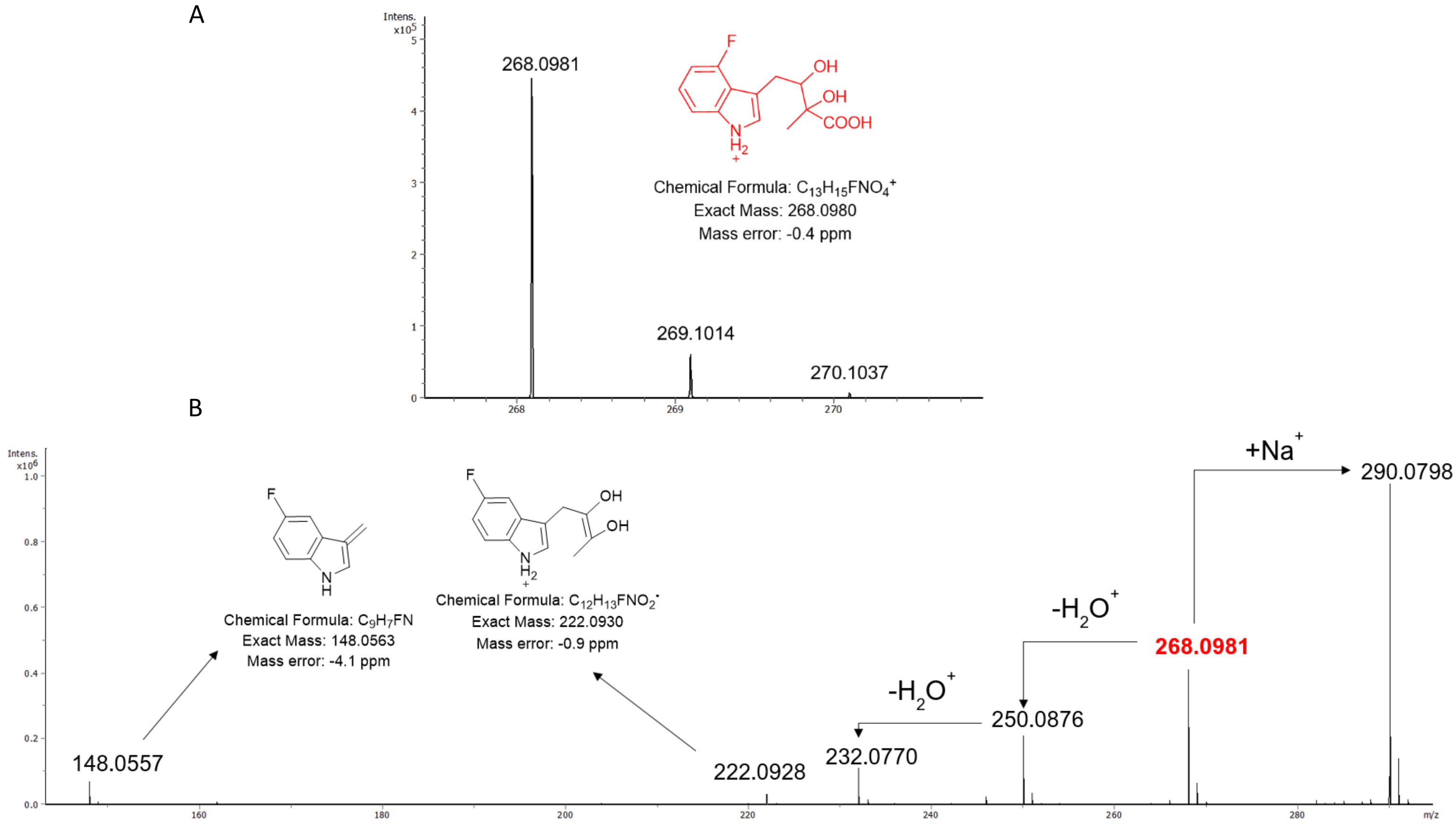

Figure S15. LC-MS (A) and MS/MS data (B) of 4-fluoro-indole-containing acyloin **31.**

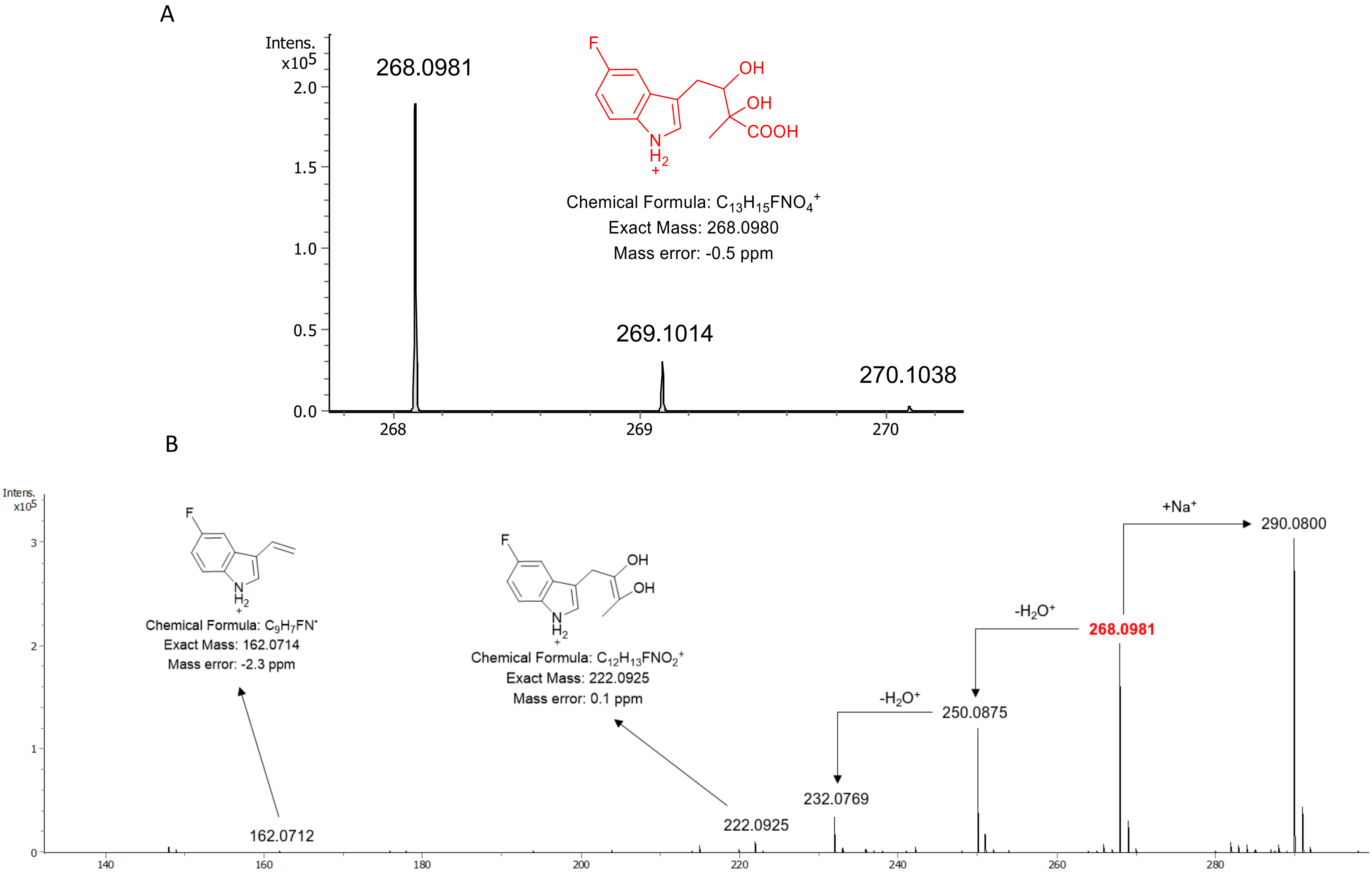

Figure S16. LC-MS (A) and MS/MS data (B) of 5-fluoro-indole-containing acyloin **32.**

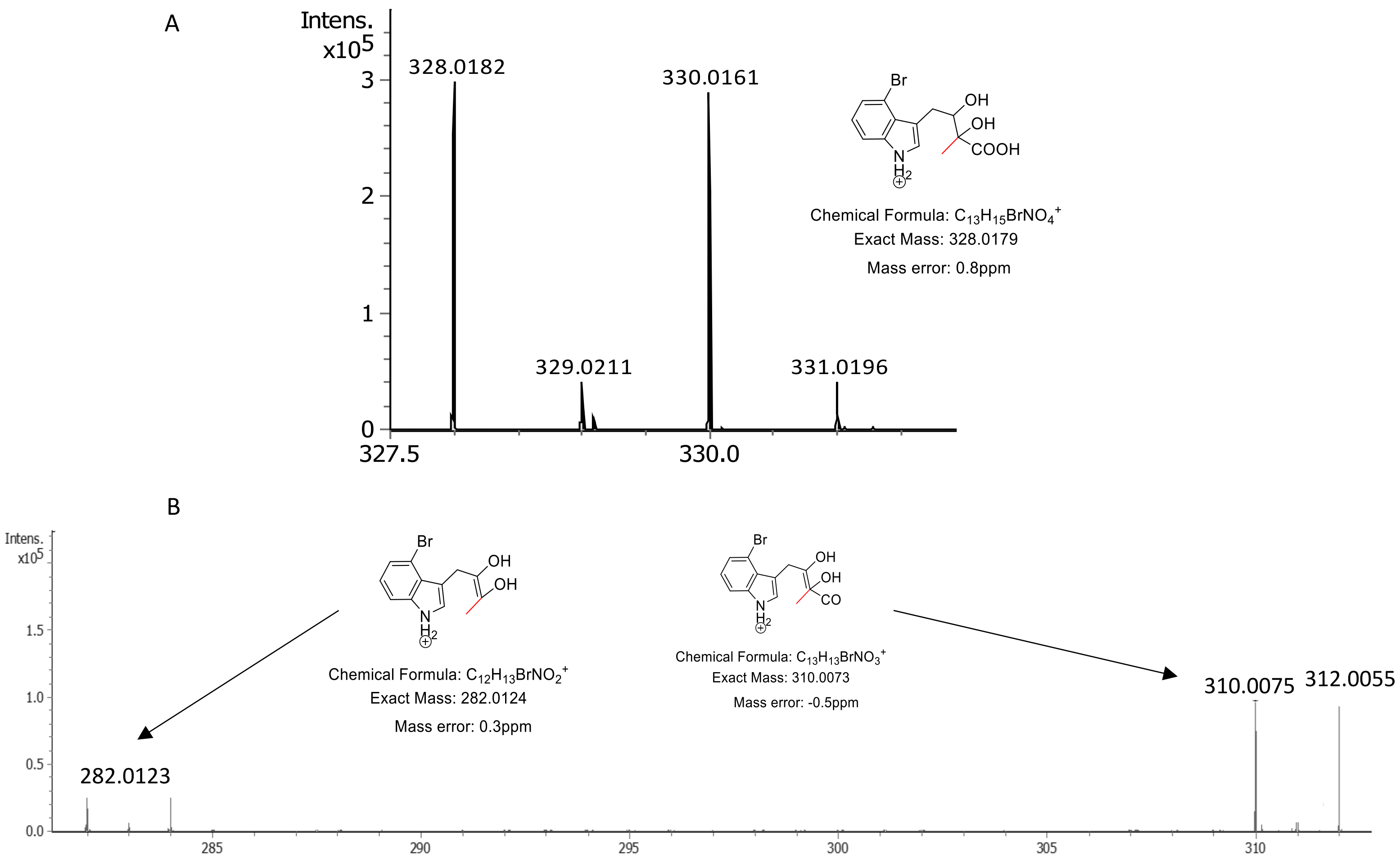

Figure S17. LC-MS (A) and MS/MS data (B) of 4-bromo-indole-containing acyloin **33**.

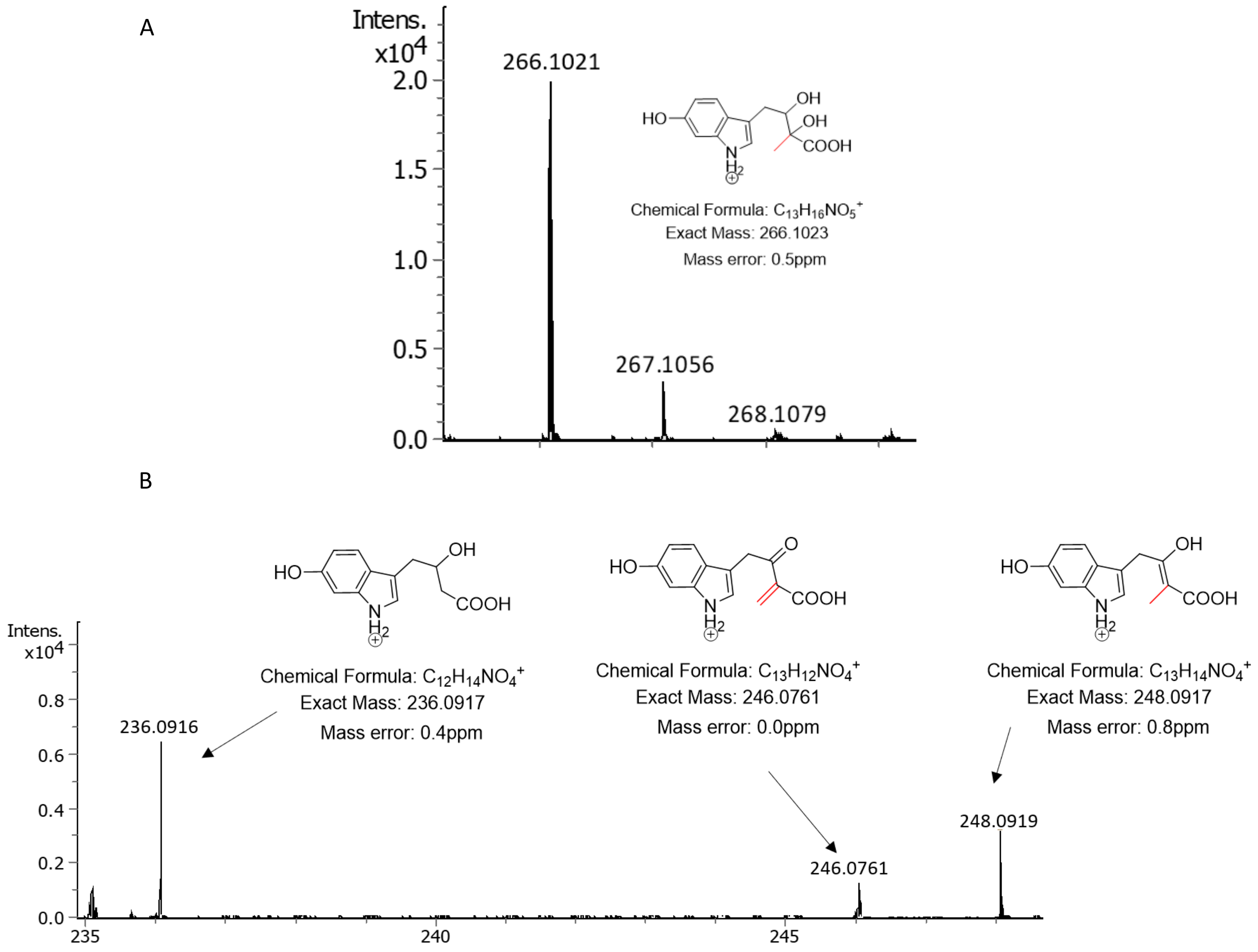

Figure S18. LC-MS (A) and MS/MS data (B) of 6-hydroxyl-indole-containing acyloin **34**.

A

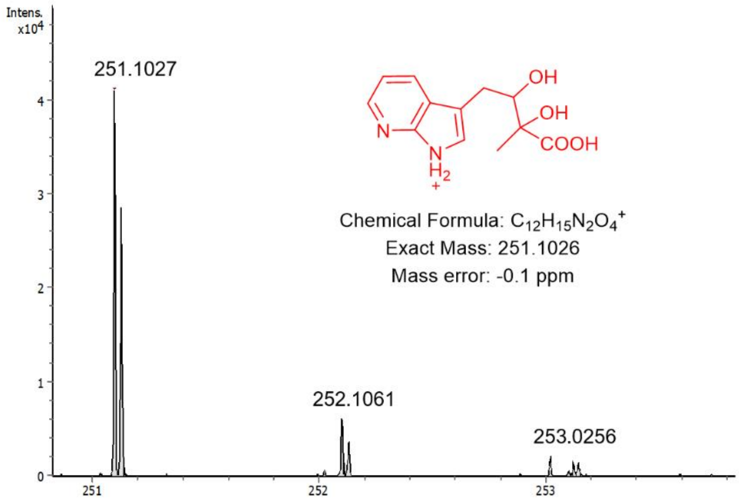

B

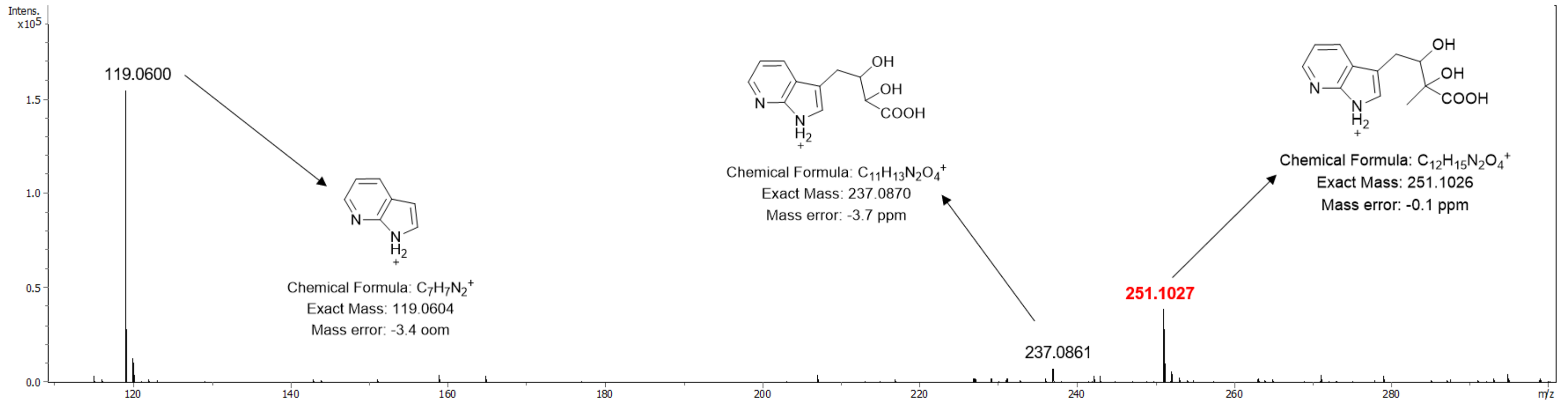

Figure S19. LC-MS (A) and MS/MS data (B) of 7-azaindole-containing-acyloin **35**.

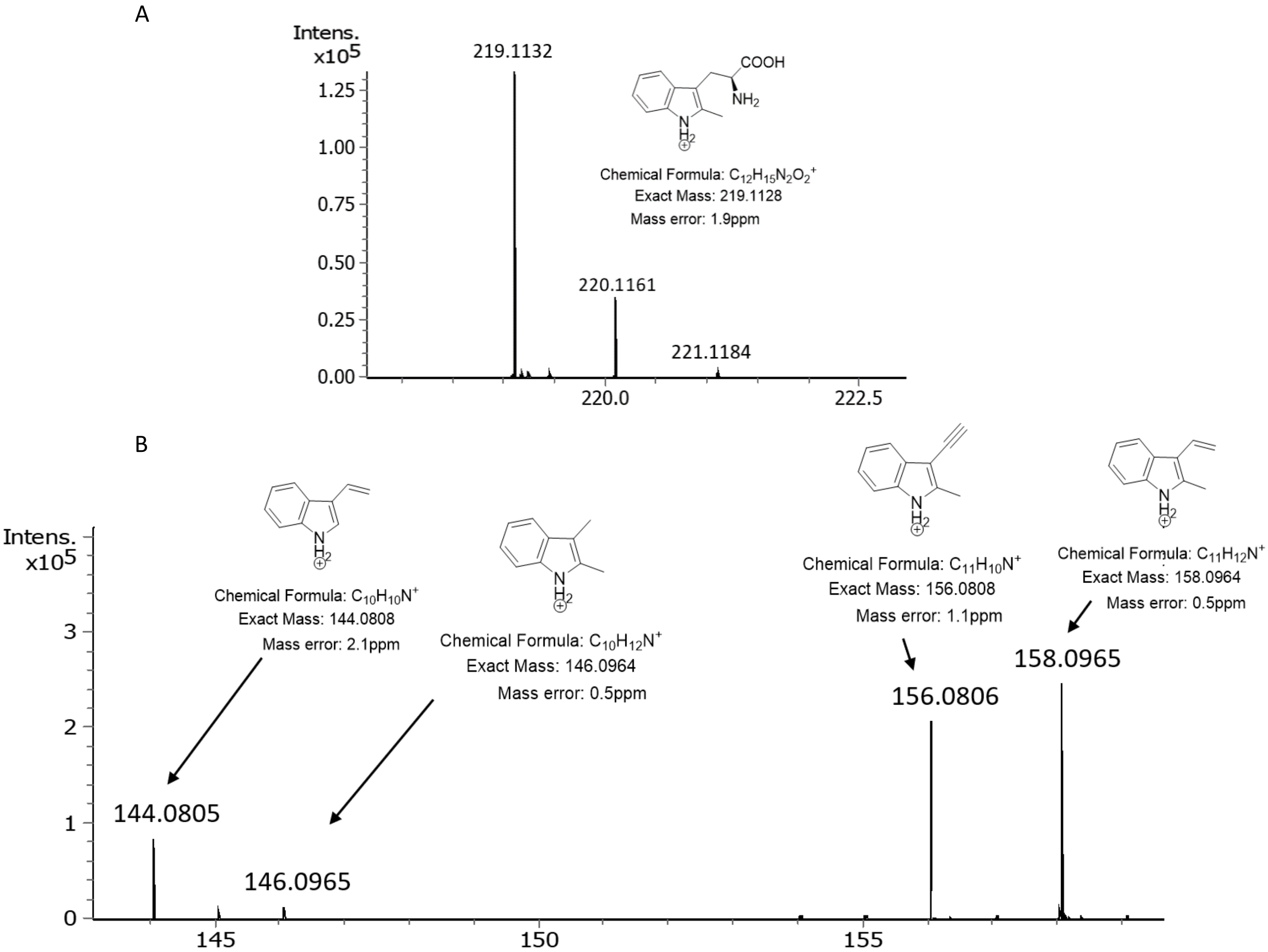

Figure S20. LC-MS (A) and MS/MS data (B) of 2-methyl-indole-tryptophan **39**.

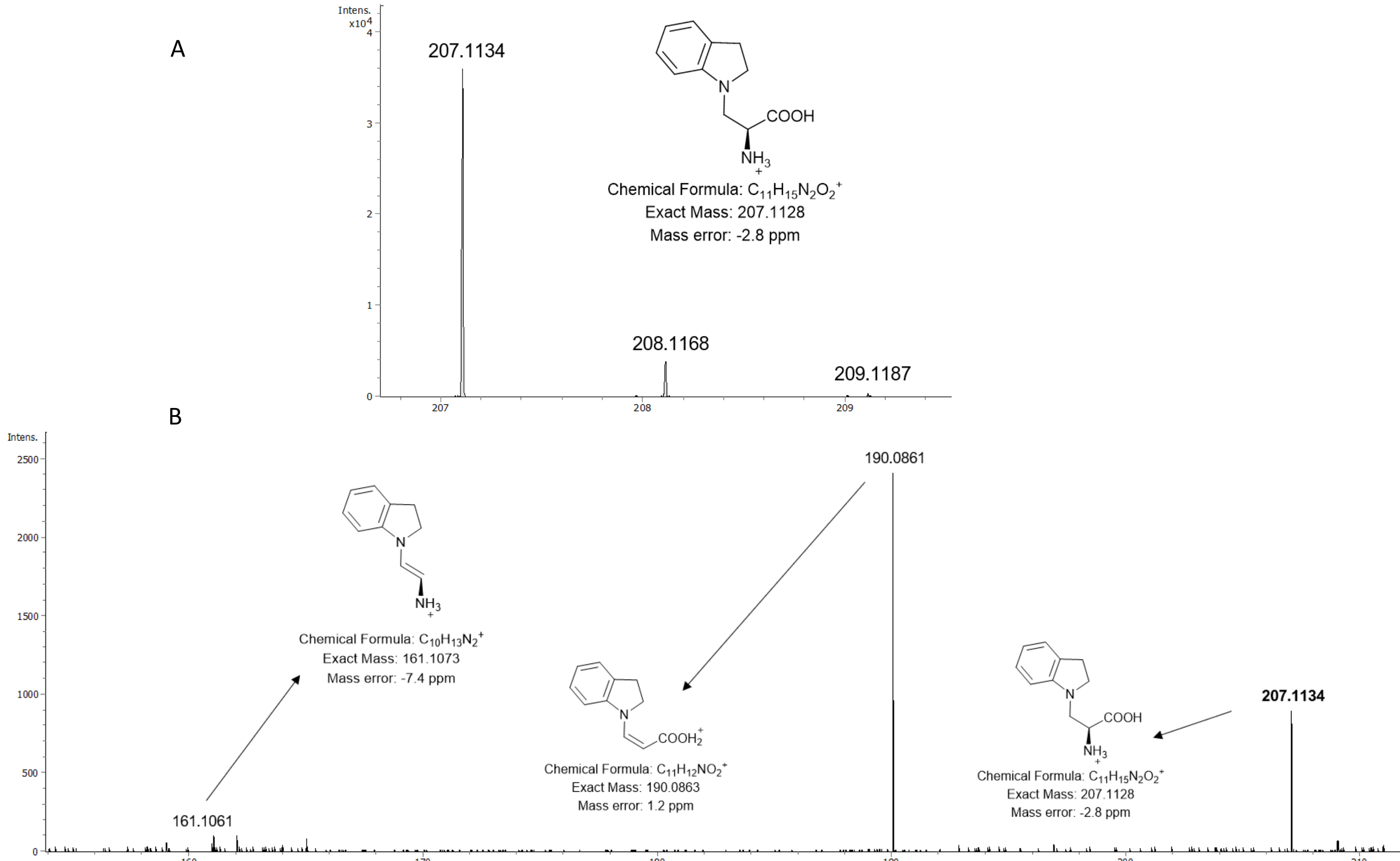

Figure S21. LC-MS (A) and MS/MS data (B) of indoline-tryptophan **40**.

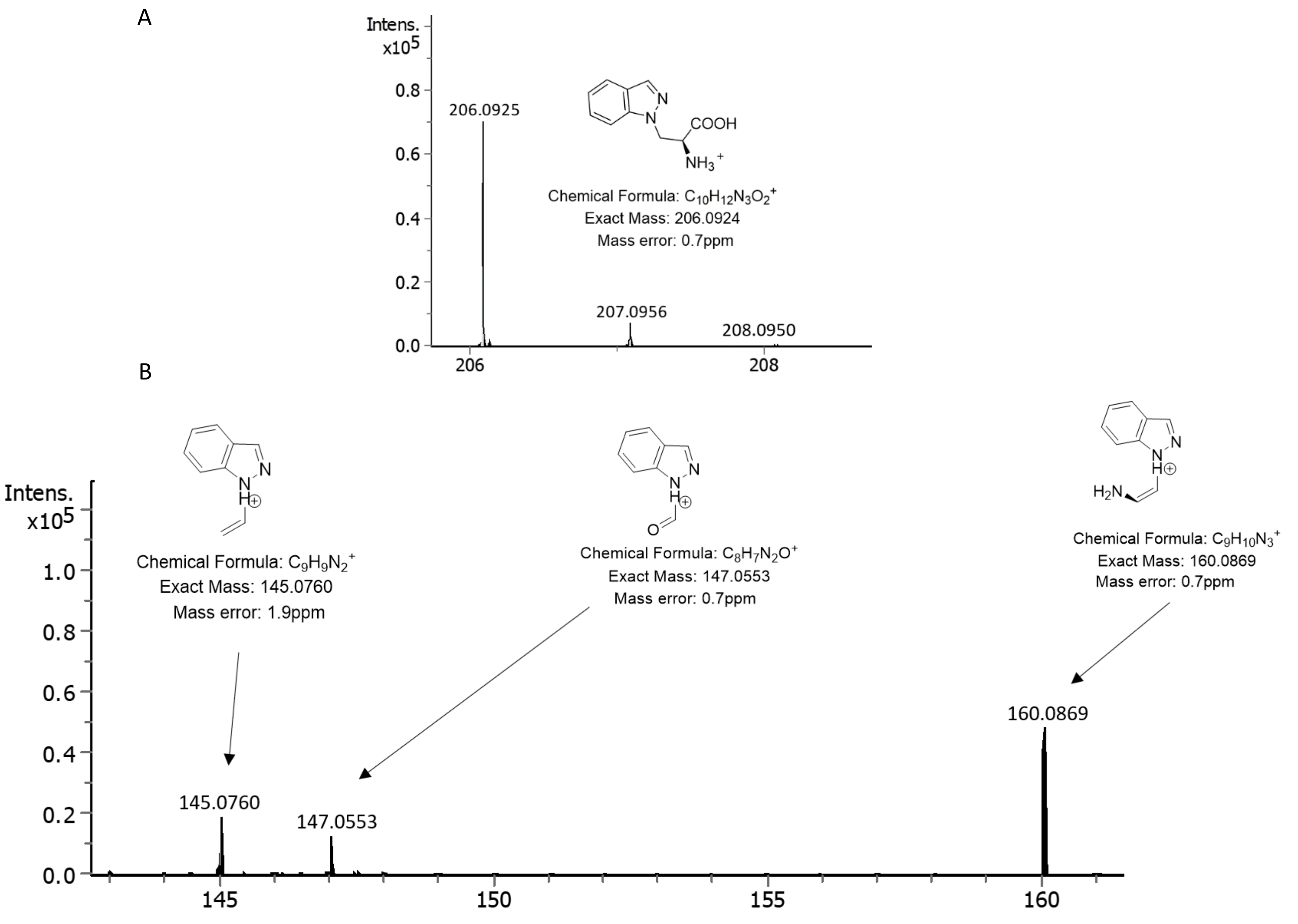

Figure S22. LC-MS (A) and MS/MS data (B) of indazole-tryptophan **41**.

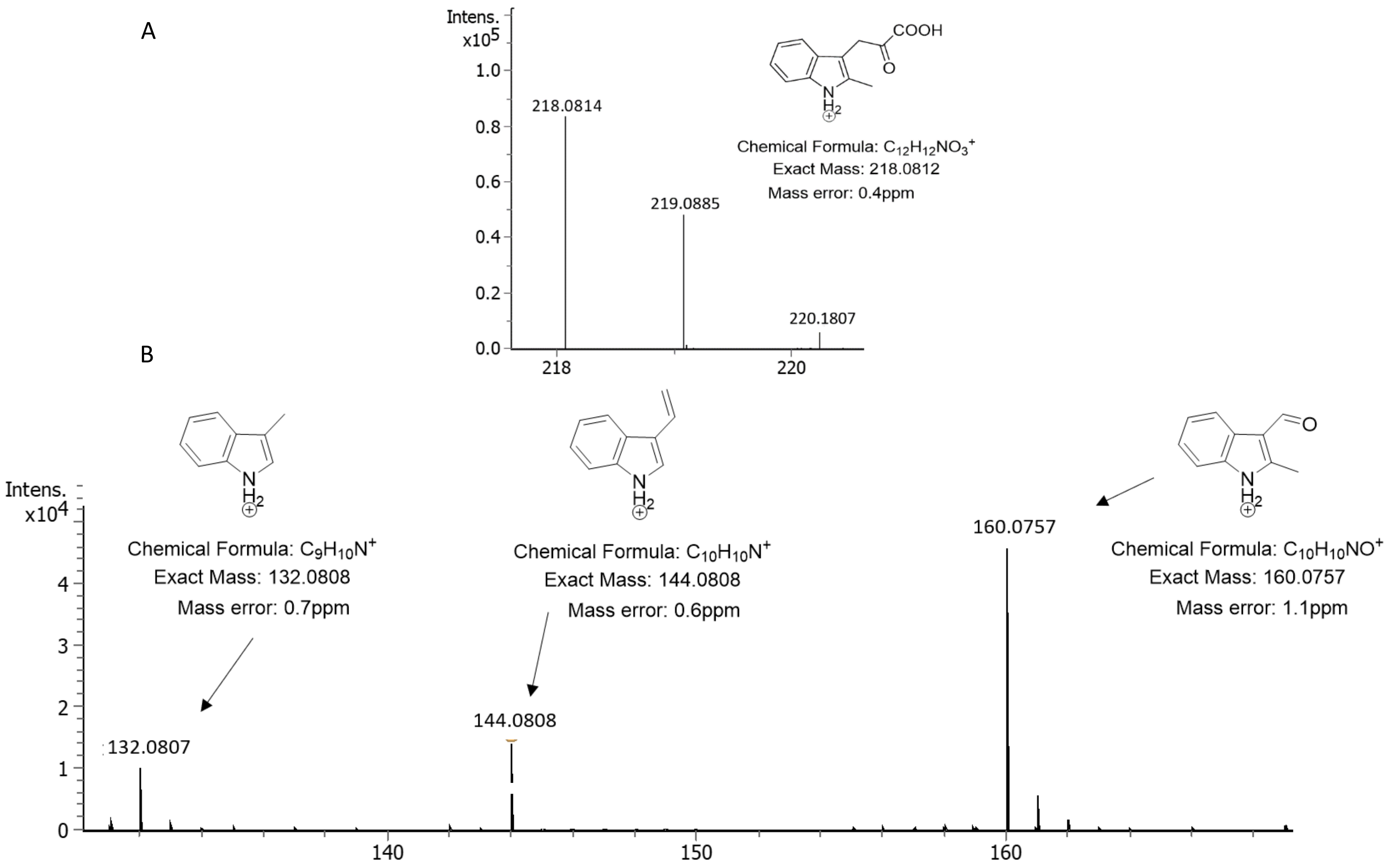

Figure S23. LC-MS (A) and MS/MS data (B) of 2-methyl-indole-3-pyruvate **42**.

A

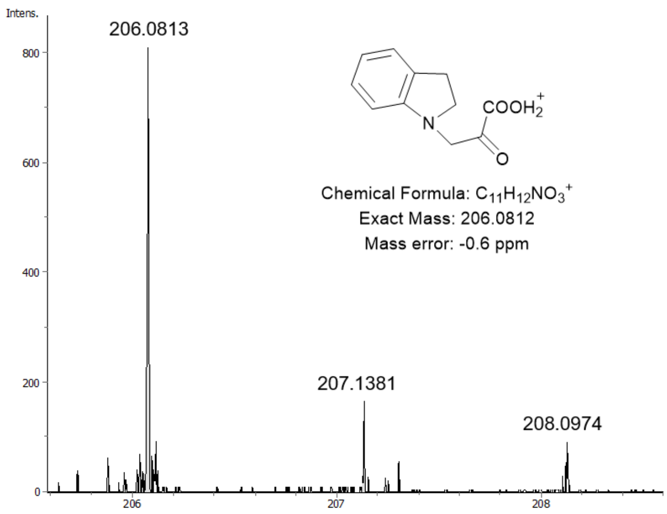

B

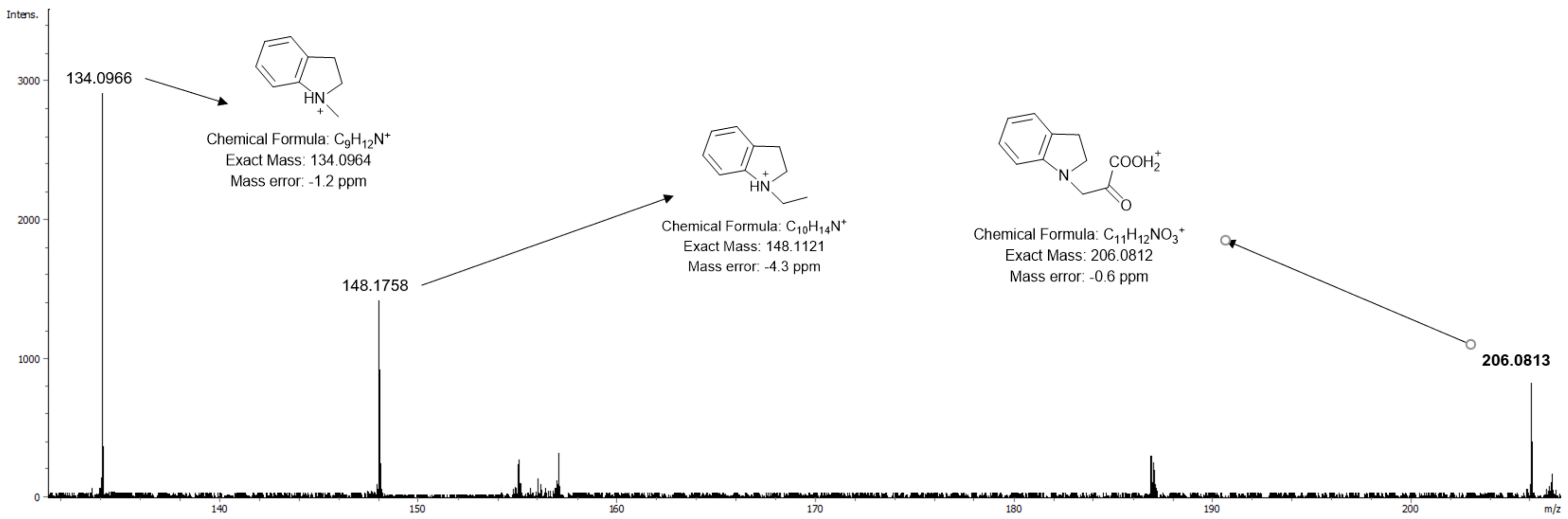

Figure S24. LC-MS (A) and MS/MS data (B) of indoline-3-pyruvate **43**.

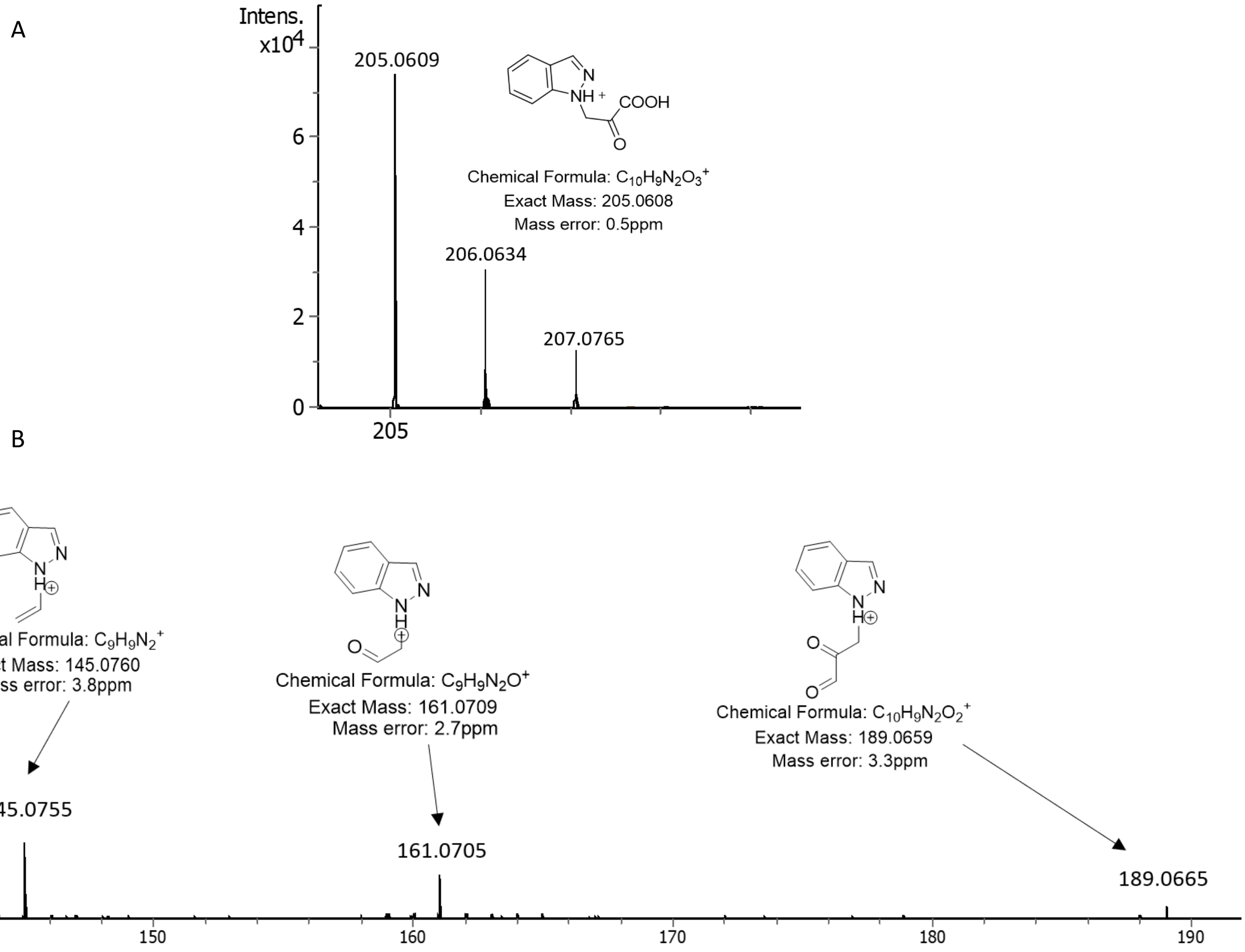

Figure S25. LC-MS (A) and MS/MS data (B) of indazole-3-pyruvate **44**.

A

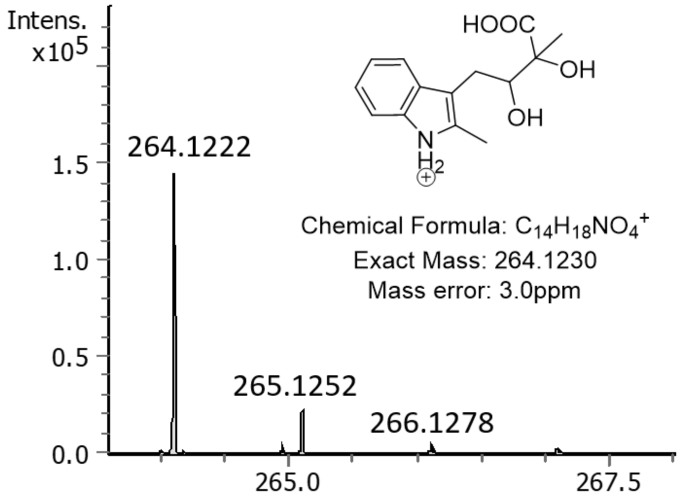

B

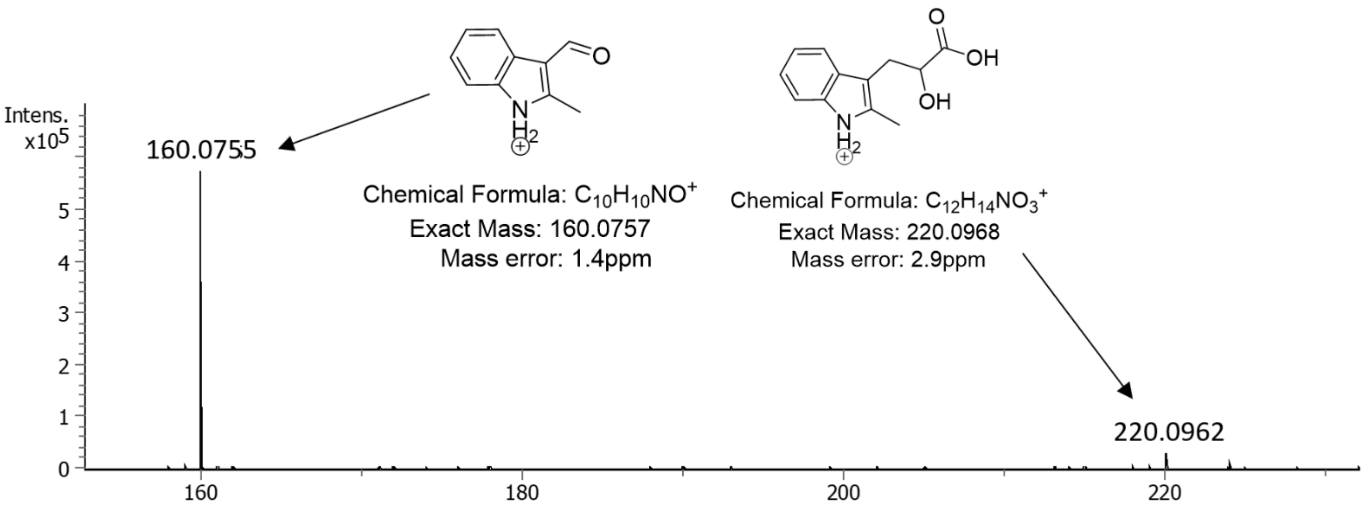

Figure S26. LC-MS (A) and MS/MS data (B) of 2-methyl-indole-containing-acyloin **45**.

A

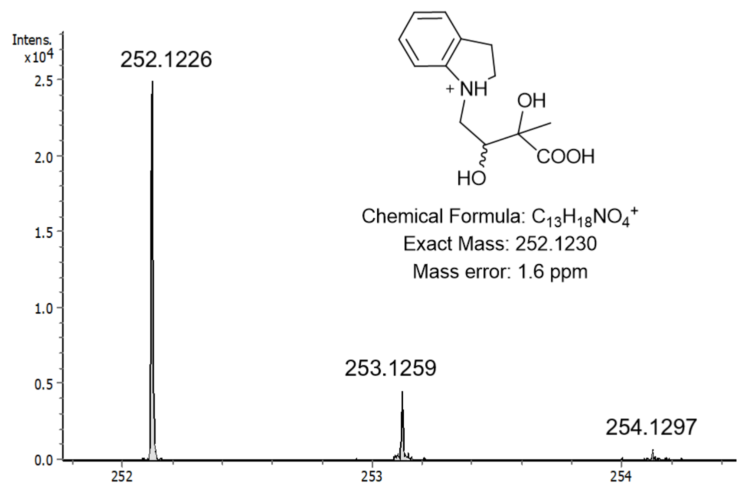

B

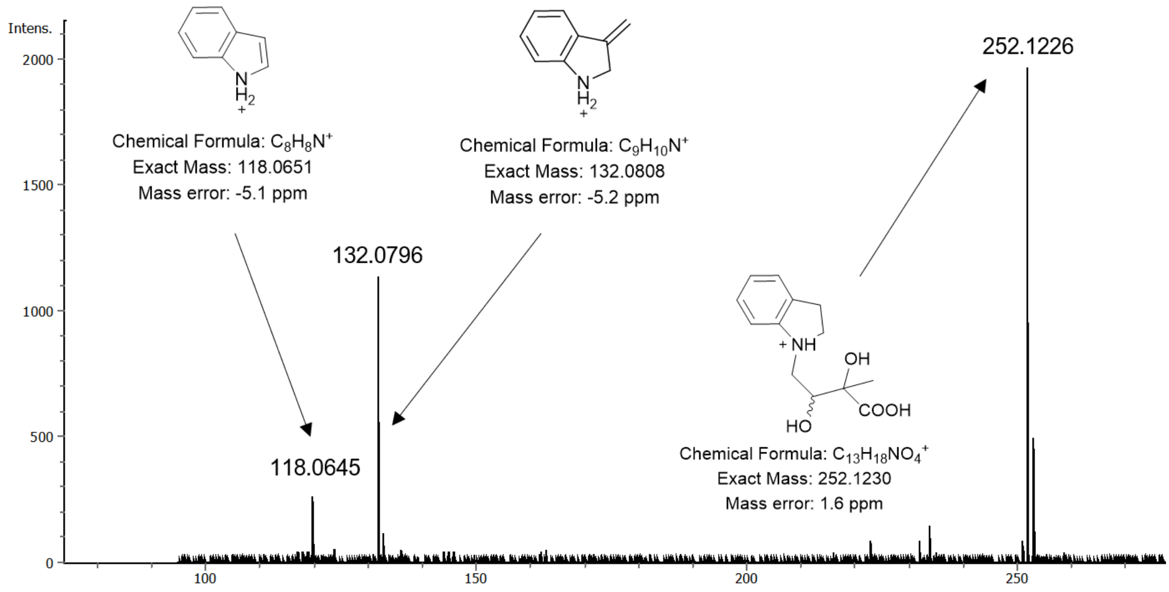

Figure S27. LC-MS (A) and MS/MS data (B) of indoline-containing-acyloin **46**.

A

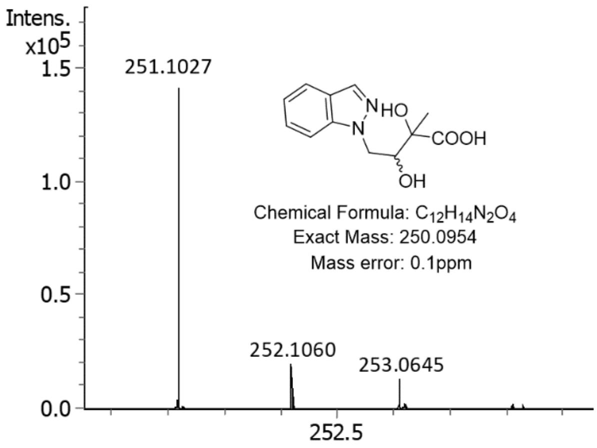

B

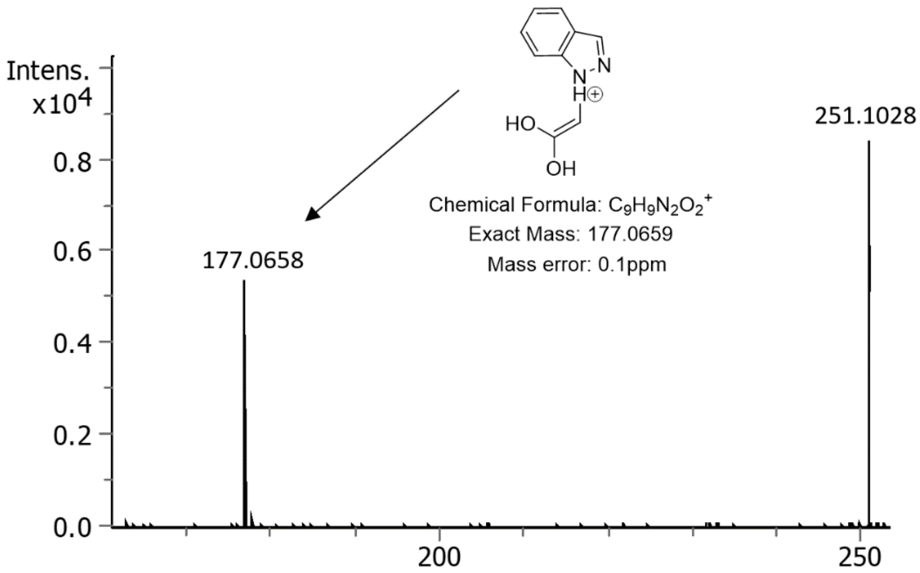

Figure S28. LC-MS (A) and MS/MS data (B) of indazole-containing-acyloin **47**.
